# Comparative transcriptomic profiling of susceptible and resistant cultivars of pigeonpea demonstrates early molecular responses during *Fusarium udum* infection

**DOI:** 10.1038/s41598-021-01587-7

**Published:** 2021-11-16

**Authors:** Arnab Purohit, Sanatan Ghosh, Shreeparna Ganguly, Madan Singh Negi, Shashi Bhushan Tripathi, Rituparna Kundu Chaudhuri, Dipankar Chakraborti

**Affiliations:** 1 Department of Biotechnology, St. Xavier’s College (Autonomous), 30, Mother Teresa Sarani, Kolkata, West Bengal 700016 India; 2 Department of Genetics, University of Calcutta, 35, Ballygunge Circular Road, Kolkata, 700019 India; 3 Sustainable Agriculture Division, TERI, India Habitat Center Complex, Lodhi Road, New Delhi, 110003 India; 4 TERI-School of Advanced Studies, 10, Institutional Area, Vasant Kunj, New Delhi, 110070 India; 5 Department of Botany, Krishnagar Govt. College, Krishnagar, West Bengal 741101 India

**Keywords:** Plant biotechnology, Plant molecular biology, Plant stress responses

## Abstract

Vascular wilt caused by *Fusarium udum* Butler is the most important disease of pigeonpea throughout the world. *F. udum* isolate MTCC 2204 (M1) inoculated pigeonpea plants of susceptible (ICP 2376) and resistant (ICP 8863) cultivars were taken at invasion stage of pathogenesis process for transcriptomic profiling to understand defense signaling reactions that interplay at early stage of this plant–pathogen encounter. Differential transcriptomic profiles were generated through cDNA-AFLP from M1 inoculated resistant and susceptible pigeonpea root tissues. Twenty five percent of transcript derived fragments (TDFs) were found to be pathogen induced. Among them 73 TDFs were re-amplified and sequenced. Homology search of the TDFs in available databases and thorough study of scientific literature identified several pathways, which could play crucial role in defense responses of the *F. udum* inoculated resistant plants. Some of the defense responsive pathways identified to be active during this interaction are, jasmonic acid and salicylic acid mediated defense responses, cell wall remodeling, vascular development and pattering, abscisic acid mediated responses, effector triggered immunity, and reactive oxygen species mediated signaling. This study identified important wilt responsive regulatory pathways in pigeonpea which will be helpful for further exploration of these resistant components for pigeonpea improvement.

## Introduction

Pigeonpea (*Cajanus cajan* (L.) Millspaugh) is an economically valuable pulse crop grown on approximately 5.62 million hectares land with 4.43 million tonnes of annual production, globally^1^. Being world’s seventh most essential grain legume, pigeonpea is an important source of edible protein and important means of financial support for the people in Asia, Eastern and Southern Africa, South America, Central America and the Caribbean countries^1^. India, the leading pigeonpea producer, is responsible for approximately 75% of the worldwide production, with a yield of 728.7 kg ha^−1^^1^. Due to several diseases and insect attack, yield of pigeonpea is poor compared to its potential yield, which is 2500 kg ha^−1^^2^. Vascular wilt disease of pigeonpea caused by soil borne fungal pathogen *Fusarium udum* Butler is the major limiting factor of pigeonpea production. Wilt occurs at all stages of plant development and results in 30–100% of yield loss depending upon the plant stage during infection^3^. *Fusarium* wilt in India causes remarkable production loss of 470,000 t of grain every year^4^.

Crop rotation, use of fungicides and development of resistant cultivars are different approaches for management of wilt. Crop rotation does not give complete and durable protection because the fungus can survive in soil for several years, while use of fungicides is not an eco-friendly or economical approach^5,6^. Therefore, development of improved cultivars with increased disease resistance is the most sustainable option to manage *Fusarium* wilt. Physiological specialization, variation in pathogenicity, location specific presence of the *F. udum* isolates and differential reactions during pathogenesis were the major limitations for breeding programs for wilt resistance^7^. *F. udum* isolates from different geographical locations of India and Kenya were divided into different pathogenic groups based on pathogenicity on different pigeonpea genotypes^7,8^. Variations were also observed during pathogenesis and establishment of wilt by different *F. udum* isolates on susceptible pigeonpea cultivar^5^. Genetic variability among *F. udum* isolates from various locations of India and Kenya were identified through DNA-based sensitive and precise methods like randomly amplified polymorphic DNA (RAPD) and amplified fragment length polymorphism (AFLP) by several researchers^5,7,8^. Additionally, out crossing nature, long life cycle and difficulty in accurate phenotyping were other obstacles of conventional resistance breeding efforts^9,10^. Genetic improvement of pigeonpea was limited due to inadequate genomic resources and low level of genetic diversity in the primary gene pool^2,11^. Complicated nature of this problem necessitates requirement of sound knowledge on molecular processes underlying resistance and susceptibility of pigeonpea cultivars for the development of cultivars with durable resistance through potent breeding programs.

Understanding the genetic basis of wilt resistance is essential for formulation of strategy to combat this disease. Earlier studies reported lipoxygenase and phytoalexins could be important biochemical markers for the development wilt resistant pigeonpea cultivars^12–14^. Molecular markers, such as RAPD^15^, simple sequence repeat (SSR)^10^ and single nucleotide polymorphism (SNP)^16^; and quantitative trait loci (QTLs)^11^ were reported to be associated with pigeonpea wilt resistance. Singh et al.^16^ used sequencing-based bulk segregant analysis to map resistance genes for *Fusarium* wilt in pigeonpea. They identified a candidate gene named *C.cajan*_03203 which codes for a retrovirus-like polyprotein. It has role in plant defense during pathogen attacks. In another study, Singh et al.^17^ deployed insertion-deletion sequencing (indel-seq) approach to identify candidate genomic regions involved in pigeonpea wilt resistance; three indels associated with wilt resistance were identified. Saxena et al.^11^ identified three QTLs (*qFW11.1, qFW11.2 and qFW11.3*) for wilt resistance in pigeonpea. Conflicting and inconsistent results suggested complexity in the inheritance of wilt resistance in pigeonpea^18,19^. Reports available on molecular markers for pigeonpea wilt resistance was very scanty; also, these reports lacked detailed information on pathogenic races/variants of *F. udum*^10^. In this scenario, detailed understanding of the molecular factors and signaling pathways behind disease susceptibility or resistance could help in development of resistant plants through different molecular breeding approaches^20–22^.

Transcriptional profiling helped to understand the defense mechanisms involved during *Fusarium* wilt resistance in plants. Variety of reactions have been observed during *Fusarium* wilt in model species, *Arabidopsis thaliana*^23^; legumes, *Cicer arietinum*^20,24^ and *Phaseolus vulgaris*^22^; and other crops like *Musa* spp.^25^ and *Cucumis melo*^21^. However, in pigeonpea, only two previous studies based on real-time PCR analysis reported roles of some specific genes (WRKY transcription factors, antioxidant enzymes and pathogenesis-related proteins) during *Fusarium* wilt^26,27^. Unfortunately, there is no report on exploring novel pathways and signaling reactions during *F. udum* induced responses in pigeonpea till date.

Complementary DNA-AFLP (cDNA-AFLP) and next generation sequencing (NGS) are convenient and effective strategies for transcriptome analysis and identification of differentially regulated genes in plants. The cDNA-AFLP method remains a robust, relatively cheap, quick, simple and reliable tool for the detection of gene expression profile. It is an ideal technique to get preliminary expression profile of plant’s responses during a plant–pathogen interaction, which could be followed up by NGS analysis to gather more information in a cost-effective way^22,28^. cDNA-AFLP was extensively applied to study differential gene expression in plants during various plant–pathogen encounters including legume–*Fusarium* interactions^22,24,29–31^.

The present study demonstrates identification of up- and down- regulated transcript derived fragments (TDFs) generated through cDNA-AFLP during early stages of wilt development in *F. udum* infected and non-infected susceptible (ICP 2376) and resistant (ICP 8863) pigeonpea cultivars. TDFs with possible roles in disease response were characterized and probable molecular interactions among those TDFs were predicted for the better understanding of defense responses during wilt development in pigeonpea.

## Results

### Morphological and anatomical changes in pigeonpea

ICP 2376 is a slow growing cultivar in comparison to ICP 8863. As a result, Noninfected ICP 2376 attained comparatively shorter height during the experimental tenure of 14–15 days (Fig. SF1a,c,e,g). Phenotypic differences were observed between MTCC 2204 (M1) inoculated susceptible ICP 2376 and resistant ICP 8863 plants. M1 infected ICP 2376 plants exhibited yellowing of roots at 30–36 h post inoculation (HPI) (Supplementary Fig. SF1b,d); yellowing and drooping of stem and leaves at 54–60 HPI (Supplementary Fig. SF1f,h); and wilting of plants occurred at 6–7 days post inoculation (DPI) (data not shown). No growth retardation or disease symptoms was observed in control plants or inoculated ICP 8863 plants even at 15 DPI.

**Figure 1:**
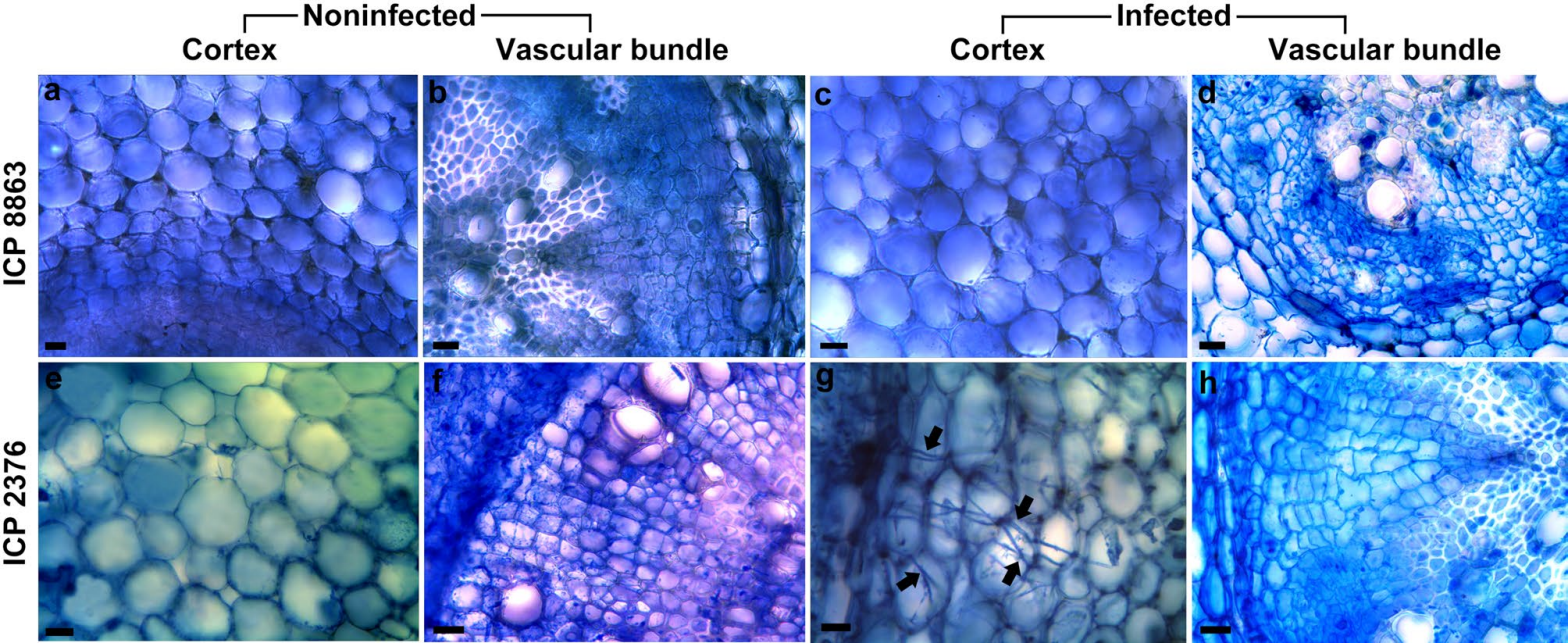
Transverse sections of control and *Fusarium udum* isolate M1 inoculated roots of pigeonpea at 36 h post inoculation. (**a**,**b**) Non-inoculated control roots of resistant ICP 8863 cultivar, (**c**,**d**) roots of ICP 8863 inoculated with M1, (**e**,**f**) non-inoculated control roots of the susceptible ICP 2376 cultivar, (**g**,**h**) roots of ICP 2376 inoculated with M1. Arrows indicate fungal mycelia. Bars represent 10 μm.

Roots of control and infected pigeonpea cultivars were taken for transverse section followed by trypan blue-lactophenol staining. *F. udum* invasion in ICP 2376 roots was observed at 30–36 HPI (Fig. 1). Two-thirds clogging of infected susceptible roots was seen at 78–84 HPI, whereas inoculated resistant plants and non-inoculated plants did not show any sign of presence of pathogen (data not shown).

### Screening of *F. udum* induced TDFs in pigeonpea through cDNA-AFLP analyses

Primarily, 66 primer combinations (E-2N × M-2N, E-3N × M-2N, E-2N × M-3N and E-3N × M-3N; N, selective nucleotides at the 3′ end of the primer; Supplementary Table S1) were used for selective amplification during cDNA-AFLP profile generation. A total of 1284 TDFs were generated; among them 1245 TDFs showed differential expression. Out of 1245 differentially expressed TDFs, 327 TDFs showed altered expression patterns due to infection. Among the 327 pathogen induced TDFs 152 showed enhanced expression and 175 TDFs showed decreased expression in resistant plants compared to susceptible plants. These bands were approximately 50–370 bp in size (including primer length).

**Figure 2:**
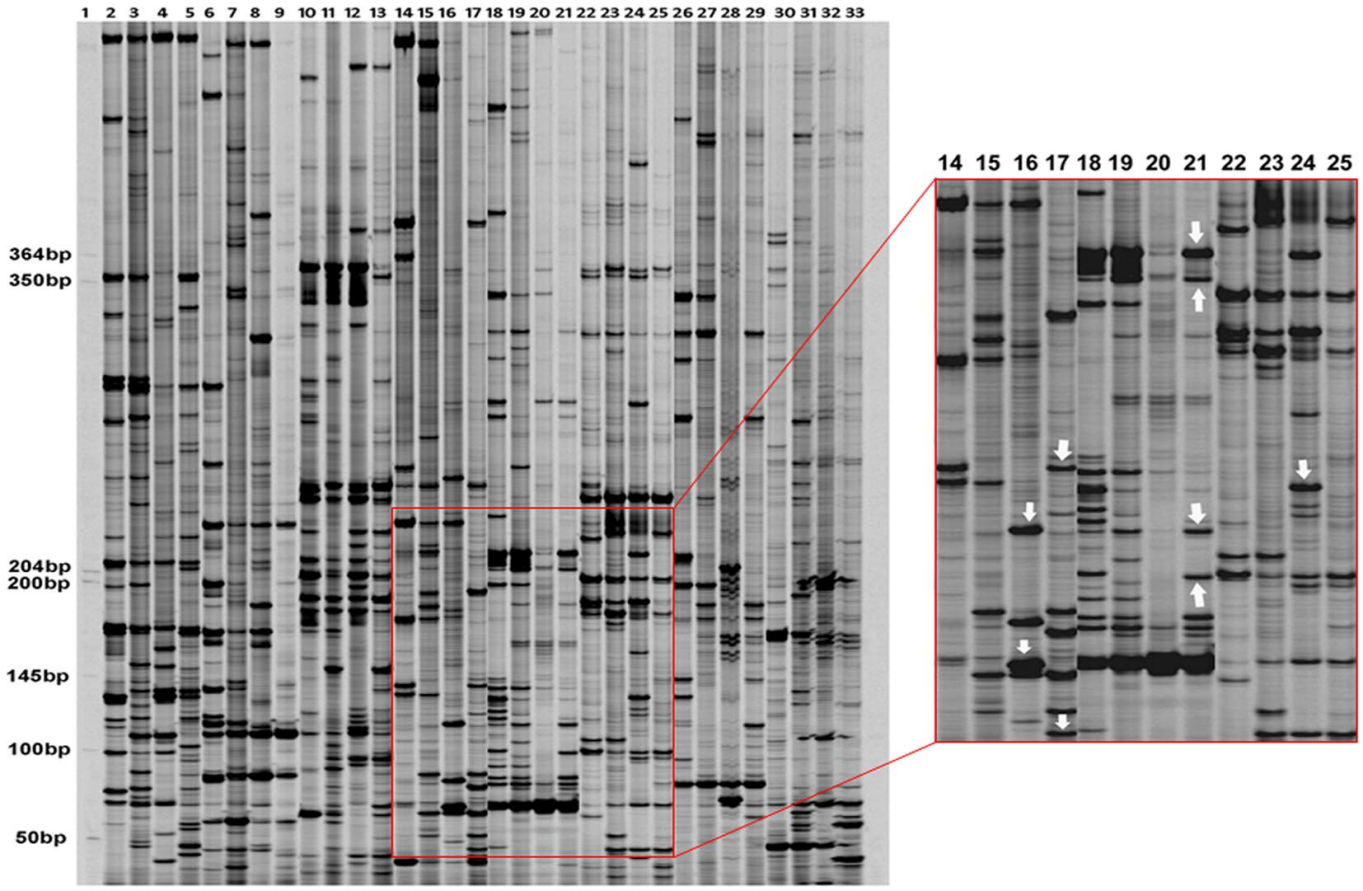
Representative cDNA-AFLP profiles of non-inoculated and *Fusarium udum* inoculated ICP 2376 and ICP 8863 samples using different primer combinations. Lanes 2, 6, 10, 14, 18, 22, 26, 30: non-inoculated control ICP 2376; lanes 3, 7, 11, 15, 19, 23, 27, 31: non-inoculated control ICP 8863; lanes 4, 8, 12, 16, 20, 24, 28, 32: inoculated ICP 2376; lanes 5, 9, 13, 17, 21, 25, 29, 33: inoculated ICP 8863; The primer combinations used were; lanes 2–5: E-AT/M-GA; lanes 6–9: E-AT/M-GT; lanes 10–13: E-AT/M-TG; lane 14–17: E-AT/M-TC; lane 18–21: E-AT/M-CA; lanes 22–25: E-AT/M-CG; lanes 26–29: E-AT/M-CAT; lanes 30–33: E-AT/M-CTA. Lane 1: 50–1500 bp size standard (LI-COR Biosciences). Inset: Representative enlarged portion of cDNA-AFLP profile to demonstrate the bands (indicated with arrows) excised for further cloning and sequencing.

**Figure 3:**
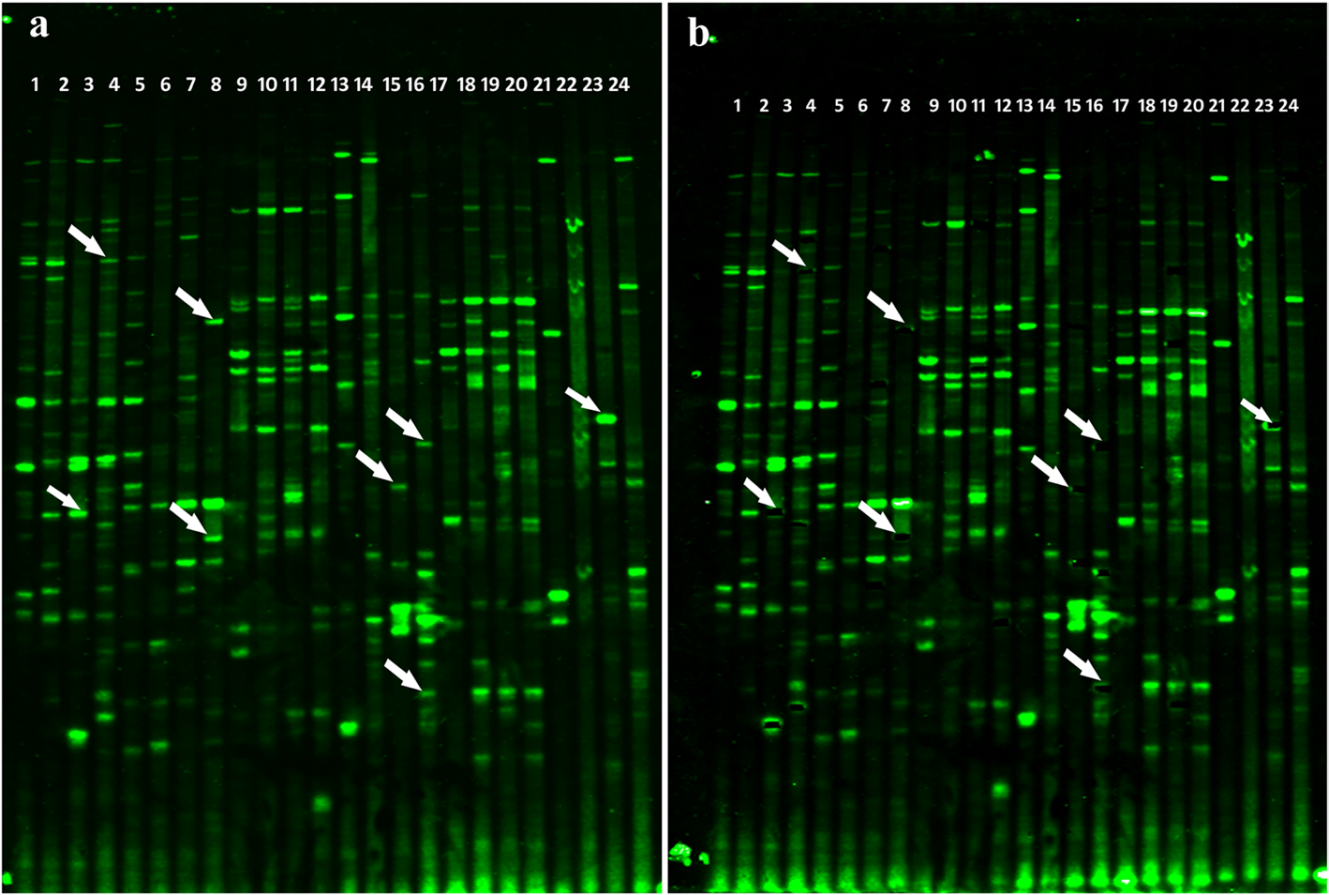
Representative cDNA-AFLP profiles of non-inoculated and *Fusarium udum* inoculated ICP 2376 and ICP 8863 samples using different primer combinations, photographed (**a**) before and (**b**) after excision of TDFs from the gel. Lanes 1, 5, 9, 13, 17, 21: non-inoculated control ICP 2376; lanes 2, 6, 10, 14, 18, 22: non-inoculated control ICP 8863; lanes 3, 7, 11, 15, 19, 23: inoculated ICP 2376; lanes 4, 8, 12, 16, 20, 24: inoculated ICP 8863; The primer combinations used were; lanes 1–4: E-AT/M-GA; lanes 5–8: E-AT/M-GT; lanes 9–12: E-AT/M-TG; lane 13–16: E-AT/M-TC; lane 17–20: E-AT/M-CG; lanes 21–24: E-ACT/M-GA. Arrows indicate some of the bands, (**a**) before and (**b**) after excision, used for further cloning and sequencing.

Among the 66 selective amplification primer combinations, 48 were selected for final cDNA-AFLP profiling on the basis of number, resolution and size of the polymorphic TDFs generated due to infection (Supplementary Table S2). In total, 1164 TDFs were generated from 48 primer combinations and 97.33% (1133) of the TDFs showed differential expression. Among these 1133 bands, 839 bands were not included in further analysis, as they either exhibited similar expression patterns in inoculated ICP 2376 and ICP 8863 plants in comparison to the control plants or showed similar profile of fold changes in expressions in both the inoculated plants in comparison to both the non-inoculated counterparts. Thus, differential expression related to dissimilarities in the genome of the two cultivars was eliminated. These 839 TDFs were not considered as pathogen induced TDFs responsible for resistance or susceptibility. Remaining 294 (25.25% of total TDFs) TDFs were uniquely present or amplified to a greater extent in infected ICP 2376/ICP 8863 plants compared to non-infected controls. These bands were treated as pathogen-induced differential TDFs associated with resistance or susceptibility and selected for further analysis. Among 294 pathogen-induced differential TDFs, 143 (48.63%) TDFs were found to show unique up-regulation or increased expression in infected ICP 8863 (resistant cultivar) compared to infected ICP 2376 (susceptible cultivar), whereas 151 (51.37%) TDFs were completely down-regulated or less expressed in infected ICP 8863 compared to infected ICP 2376 (Supplementary Table S2, Supplementary Fig. SF2). One hundred and nine distinctly up- and down-regulated TDFs in the range of approximately 80–370 bp (including primer length) were cut from the dried urea polyacrylamide gel electrophoresis (urea-PAGE) gel. Photograph was taken again to confirm recovery of the correct bands from the gel (Figs. 2 and 3, Supplementary Fig. SF3 and SF4). A total of 73 TDFs which were > 50 bp in length (excluding primer length) were sequenced after cloning in the pGEM-T Easy Vector.

### Bioinformatics analysis of pathogen induced differential TDFs

Among 73 sequenced TDFs, 9 TDFs were repetition of similar genes or transcripts, which were excluded from the study. Sixty four unique TDFs with sizes ranging from 50 to 336 bp (excluding primer length) were analyzed for homology with known or predicted genes. Six TDFs were similar to fungal proteins, and were also not included in further analysis (Supplementary Table S3). Remaining 58 TDFs were differentially regulated in susceptible and resistant plants due to infection (Supplementary Table S4). Ten of those 58 sequences showed no hit or less similar to known or predicted genes from pigeonpea, legumes or any other plants during the search in three databases (National Center for Biotechnology Information (NCBI)^32^, Legume information system (LIS)^33^ and Phytozome^34^).

The remaining 48 TDFs showed high similarities (97–100%) in the NCBI and LIS database search. The sequence homology with best *E* values was further investigated for functional annotation with the help of available literature and Uniprot website^35^ (https://www.uniprot.org) (Table 1). Among 48 TDFs, 37 showed homology to known or predicted *C. cajan* genes with putative functions whereas 11 TDFs were similar to uncharacterized or unannotated *C. cajan* sequences. Uncharacterized and unannotated TDFs homologous to pigeonpea sequences were tried to match with other legume (Fabaceae) gene sequences with known functions. Five of those 11 TDFs were annotated to other legumes with known functions. These five annotated pigeonpea TDFs with known functions belonged to *Vigna radiata* (2), *Lupinus angustifolius* (1), *Abrus precatorius* (1) and *Glycine max* (1). Among the mentioned 5 annotated TDFs, 90–99% homology was found for 2 TDFs (*G*. *max, A*. *precatorius*), 80–89% for 2 TDFs (*V. radiata* and *L. angustifolius*) and 79% for 1 TDF (*V. radiata*). Altogether 42 TDFs were used for functional interpretation.

**Table 1:** Differentially expressed pathogen induced transcript derived fragments (TDFs) revealed by BLASTN search.

Cloned TDFs	Differential expression^1^ (inoculated ICP 2376-inoculated ICP 8863)	Size of TDFs (bp)	Homology (NCBI^2^), (LIS^3^)	Function of TDFs	Query cover	*E*-value	Identity	Location at chromosome no	Accession of the NCBI hit	Accession of submitted TDFs
PF1U1	A–P	336	*Vigna radiata* var. radiata VQ motif-containing protein 4-like	Stress	74%	7e−63	79%	10	XM_014637099.1	MF621019
PF1U2	A–P	190	*Cajanus cajan* immune-associated nucleotide-binding protein 9-like (Alternative name: AIG1-like protein)	Stress	100%	1e−88	99%	11	XM_020369713.1	MF621020
PF1U3	A–P	168	*Cajanus cajan* F-box/kelch-repeat protein At1g67480	Secondary Metabolism	100%	5e−79	100%	Unplaced/Unlocalized scaffolds	XM_020374267.1	MF624632
PF1U4	A–P	59	No match	NA^4^	NA	NA	NA	NA	NA	MF661776
PF1D6	P–A	59	*Cajanus cajan* vesicle-associated protein 2–2-like	Intracellular transport	100%	3e−23	100%	2	XM_020379397.1	MH188930
PF2U8	A–P	127	*Dictyostelium discoideum* AX4 ABC transporter-related protein (abcF3)	Transport/Vascular Development	99%	3e−11	75%	NA	XM_638524.1	MF737363
PF2U9	A–P	60	*Cajanus cajan* epidermis-specific secreted glycoprotein EP1	Water transport	100%	4e−21	100%	Unplaced	XM_020375752.1	MF661777
PF2D10	P–A	268	*Cajanus cajan* squamosa promoter-binding-like protein 16	Transcription factor	100%	5e−133	100%	11	XM_020372438.1	MF661778
PF2D12	P–A	102	*Cajanus cajan* insulin-degrading enzyme-like 1, peroxisomal	Wound	77%	4e−30	97%	3	XM_020357222.1	MH188931
PF3D15	P–A	199	*Cajanus cajan* E3 ubiquitin-protein ligase RDUF2-like	Secondary Metabolism	93%	5e−87	99%	4	XM_020359198.1	MF684635
PF3D16	P–A	110	*Cajanus cajan* cysteine protease RD19A-like	Stress	100%	9e−48	100%	Unplaced	XM_020381718.1	MF684636
PF4U17	A–P	216	*Cajanus cajan* 40S ribosomal protein S25	Protein Metabolism	100%	3e−102	99%	6	XM_020361628.1	MF684637
PF4U18	A–P	121	*Cajanus cajan* WUSCHEL-related homeobox 4-like	Vascular Development	100%	7e−95	100%	Unplaced	XM_020351983.1	MF774337
PF4D21	P–A	139	*Cajanus cajan* transcription factor MYB46-like	Vascular Development	100%	1e−70	100%	Unplaced	XM_020377594.1	MF774338
PF4D22	P–A	71	*Cajanus cajan* patatin-like protein 6	Hydrolase activity/defense response	100%	3e−30	100%	2	XM_020347638.1	MH188932
PF6U27	A–P	258	*Cajanus cajan* MLO-like protein 1	Stress	100%	6e−125	99%	Unplaced	XM_020352730.1	MF684638
PF6D28	P–A	85	No match	NA	NA	NA	NA	NA	NA	MF684639
PF7D29	P–A	266	No match	NA	NA	NA	NA	NA	NA	MF684640
PF7D30	P–A	107	No match	NA	NA	NA	NA	NA	NA	MH188933
PF8U31	A–P	193	*Cajanus cajan*; Scaffold000321 (LIS)	NA	NA	7.67704e−85	178/180 (98.9%)	Unplaced	NA	MF737365
PF8D32	P–A	149	*Tetrahymena thermophila* SB210 tubulin partial mRNA	Cell component	69%	7e−26	88%	NA	XM_001023006.3	MF737364
PF8D33	P–A	62	*Cajanus cajan*; Cc07 (LIS)	NA	NA	4.77033e−20	60/62 (96.8%)	7	NA	MH188934
PF9U34	A–P	246	*Cajanus cajan* phytochrome A	Circadian rhythm	100%	8e−117	99%	11	XM_020370451.1	MF684641
PF9U35	A–P	185	*Cajanus cajan* tubulin alpha-3 chain	Cell component	100%	3e−88	100%	Unplaced	XM_020374014.1	MF684642
PF9U36	A–P	99	*Lupinus angustifolius* general negative regulator of transcription subunit 3-like	Signal transduction	94%	2e−16	83%	7	XR_002108976.1	MF684643
PF10U37	A–P	313	*Cajanus cajan* peroxidase 73-like	Stress	100%	1e−154	99%	11	XM_020373235.1	MF684644
PF10D39	P–A	135	*Cajanus cajan* WAT1-related protein At4g28040-like	Transport	99%	2e−67	100%	Unplaced	XM_020375749.1	MH188935
PF11U40	A–P	231	*Cajanus cajan* auxin efflux carrier component 4-like (Gene name: *PIN4*)	Vascular Development	100%	2e−120	99%	Unplaced	XM_020351624.1	MF774339
PF12U41	A–P	230	*Cajanus cajan* ubiquitin-conjugating enzyme E2 7-like	Protein metabolism	100%	7e−111	99%	10	XM_020367914.1	MF684645
PF13U42	A–P	63	*Cajanus cajan* NADP-specific glutamate dehydrogenase-like	Metabolism	100%	1e−22	100%	11	XM_020370640.1	MF684646
PF14U44	A–P	81	*Cajanus cajan* auxin efflux carrier component 2 (Gene name: *PIN2*)	Vascular Development	100%	9e−35	100%	Unplaced	XM_020374647.1	MF774340
PF14D45	P–A	228	*Cajanus cajan* pathogenesis-related protein PR-1-like (LOC109809019), mRNA	Stress	100%	2e−111	100%	11	XM_020372239.1	MH188936
PF15D47	P–A	113	*Abrus precatorius* phosphatidylglycerophosphatase GEP4, mitochondrial	Metabolism/Cardiolipin biosynthetic process	100%	9e−37	92.04%	2	XM_027488693.1	MH188937
PF16D52	P–A	203	*Cajanus cajan* probable WRKY transcription factor 70	Transcriptional regulation/defense	100%	7e−98	100%	Unplaced	XM_020353925.1	MH188938
PF17U54	A–P	52	*Cajanus cajan* expansin-like B1	Cell wall organization	100%	4e−19	100%	Unplaced	XM_020349562.1	MH188939
PF17D57	P–A	103	*Cajanus cajan*; Cc02 (LIS)	NA	NA	4.05941e−48	102/103 (99%)	2	NA	MH188940
PF18U59	A–P	124	*Glycine max* CLE24 protein gene (CLE: CLAVATA3/ESR)	Vascular Development	95%	3e−43	94.96%	Unplaced	HM585122.1	MF774341
PF18U60	A–P	50	*Cajanus cajan* casein kinase II subunit alpha-2	Ribosome biogenesis/Circadian rhythm	100%	1e−15	98%	3	XM_020356227.1	MF737366
PF19U63	A–P	285	*Cajanus cajan* receptor homology region, transmembrane domain- and RING domain-containing protein 2-like	protein transport	100%	3e−142	100%	Unplaced	XM_020376063.1	MF684647
PF19U65	A–P	92	No match	NA	NA	NA	NA	NA	NA	MF684648
PF19U66	A–P	65	*Cajanus cajan* methionine gamma-lyase-like	isoleucine biosynthesis	100%	1e−23	100%	2	XM_020353840.1	MF684649
PF20U69	A–P	113	*Cajanus cajan* SUPPRESSOR OF ABI3-5	Drought tolerance	100%	9e−48	99%	11	XM_020373142.1	MF684650
PF22U72	A–P	246	*Cajanus cajan* importin subunit beta-1-like	Protein transport	100%	4e−121	100%	Unplaced	XM_020380243.1	MF684651
PF22U73	A–P	126	*Vigna radiata* var. radiata zinc finger protein CONSTANS-LIKE 1-like	Transcription factor	100%	4e−28	81%	6	XM_014664974.1	MF737356
PF22D74	P–A	240	Cajanus cajan uncharacterized LOC109802437, ncRNA	NA	100%	3e−116	99%	7	XR_002240099.1	MH188941
PF23D78	P–A	87	No match	NA	NA	NA	NA	NA	NA	MH188942
PF25U81	A–P	209	*Cajanus cajan* disease resistance protein RPM1-like	defense	100%	4e−101	100%	Unplaced	XM_020384690.1	MH188943
PF25D85	P–A	87	*Cajanus cajan*; Scaffold000118 (LIS)	NA	NA	5.95362e−39	86/87 (98.9%)	Unplaced	NA	MH188944
PF28D89	P–A	77	*Cajanus cajan* nudix hydrolase 15, mitochondrial-like	Metabolism	98%	2e−29	100%	Unplaced	XM_020349482.1	MH188945
PF29D92	P–A	119	*Cajanus cajan* ABC transporter G family member 11-like	Vascular Development	100%	1e−58	100%	9	XM_020366663.1	MF774342
PF31U96	A–P	61	*Cajanus cajan* BRCA1-associated protein	DNA repair	100%	1e−21	100%	11	XM_020372094.1	MF737357
PF31U97	A–P	56	*Cajanus cajan* histidine-containing phosphotransfer protein 1-like	Hormone signal transduction	100%	6e−19	100%	2	XM_020354445.1	MF737358
PF32U99	A–P	71	*Cajanus cajan* cationic peroxidase 1-like	Stress	100%	7e−27	100%	2	XM_020352392.1	MF737359
PF32D101	P–A	146	*Cajanus cajan* scarecrow-like protein 21	Transcriptional regulation/During defence	100%	4e−67	100%	Unplaced	XM_020351051.1	MF737360
PF33U103	A–P	242	No match	NA	NA	NA	NA	NA	NA	MF737361
PF35U107	A–P	52	*Cajanus cajan*; Scaffold133013 (LIS)	NA	NA	8.45481e−14	48/52 (92.3%)	Unplaced	NA	MF737367
PF35U108	A–P	50	No match	NA	NA	NA	NA	NA	NA	MF737368
PF35D109	P–A	221	*Cajanus cajan* protein XRI1	DNA repair	96%	3e−102	99%	9	XM_020367288.1	MF737362

^1^A refers to down-regulation and P refers to up-regulation, ^2^*NCBI* National Center for Biotechnology Information, ^3^*LIS* Legume Information System, ^4^*NA* not applicable.

Up- and down-regulated 42 gene fragments similar to known or predicted legume genes with known functions were related to stress- or defense-response (TDFs similar to VQ motif-containing protein 4, AIG1, cysteine protease RD19A, MLO-like protein 1, peroxidase 73, pathogenesis-related protein PR-1, SUPPRESSOR OF ABI3-5, disease resistance protein RPM1, and cationic peroxidase 1), wound-response (TDFs similar to insulin-degrading enzyme-like 1), cell wall organization (TDFs similar to expansin-like B1), transport (TDFs similar to vesicle-associated protein 2-2, epidermis-specific secreted glycoprotein EP1, WAT1-related protein At4g28040, receptor homology region, transmembrane domain- and RING domain-containing protein 2, and importin subunit beta-1), signal transduction (TDFs similar to histidine-containing phosphotransfer protein 1), metabolism (TDFs similar to F-box/kelch-repeat protein, E3 ubiquitin-protein ligase RDUF2, 40S ribosomal protein S25, patatin-like protein 6, ubiquitin-conjugating enzyme E2 7, NADP-specific glutamate dehydrogenase, phosphatidylglycerophosphatase GEP4, methionine gamma-lyase, and nudix hydrolase 15), vascular development (TDFs similar to WUSCHEL-related homeobox 4, transcription factor MYB46, auxin efflux carrier component 2 (PIN2), auxin efflux carrier component 4 (PIN4), CLAVATA3/ESR-related 24 (CLE24) protein gene, and ABC transporter G family member 11), transcription factors and transcription regulation (TDFs similar to squamosa promoter-binding-like protein 16, WRKY transcription factor 70, zinc finger protein CONSTANS-LIKE 1, scarecrow-like protein 21 and general negative regulator of transcription subunit 3), DNA repair (TDFs similar to BRCA1-associated protein and protein XRI1), circadian rhythm (TDFs similar to phytochrome A and casein kinase II subunit alpha-2), and cell component (TDFs similar to tubulin alpha-3 chain) (Supplementary Table S3, Supplementary Fig. SF5). Detailed analysis of those TDFs using scientific literature and KEGG pathway database^36^ was performed.

48 TDFs similar to pigeonpea genes were found in different genomic locations. Twenty seven TDFs were distributed in chromosomes 2, 3, 4, 6, 7, 9, 10, and 11; and 21 TDFs were found in unplaced/unlocalized scaffolds. Eight and 7 TDFs were found in chromosomes 11 and 2, respectively; 3 TDFs were present on chromosome 7; 2 TDFs were found on each of chromosomes 3, 6, 9 and 10 and 1 on chromosome 4 (Table 1).

Accession numbers of the 58 sequences submitted in NCBI are mentioned in Table 1.

### Analysis of TDFs by semi-quantitative reverse transcriptase PCR

**Figure 4:**
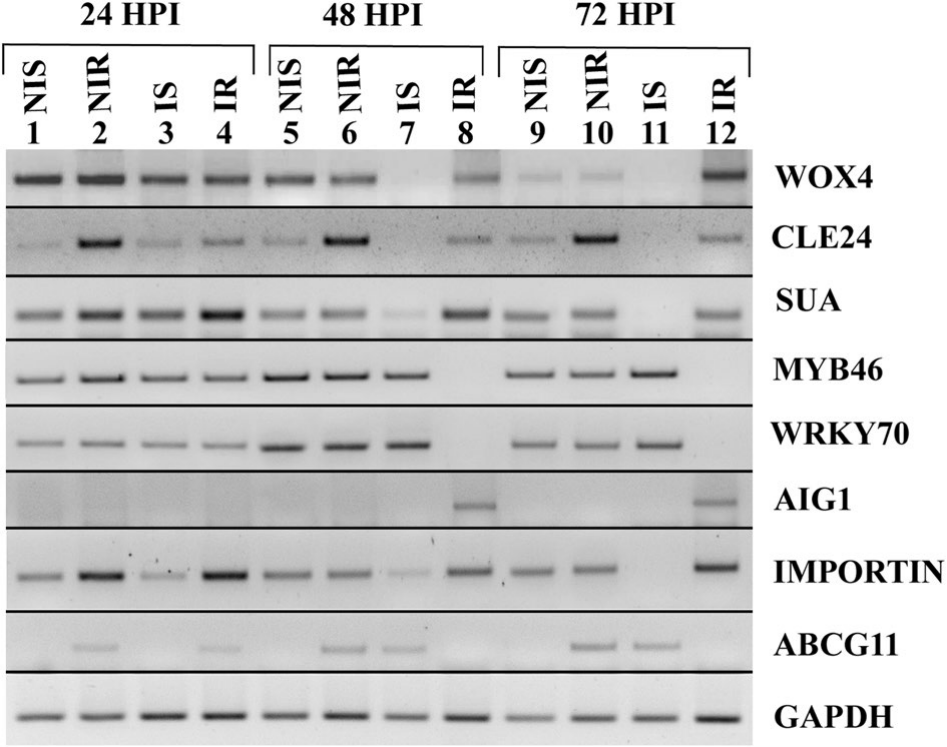
Semi-quantitative reverse-transcriptase PCR amplification patterns of selected genes in the control and *Fusarium udum* inoculated susceptible ICP 2376 and resistant ICP 8863 pigeonpea cultivars at three different time points in 2% agarose gel. Lanes 1, 5 and 9: TDFs derived from non-inoculated susceptible ICP 2376 (NIS) at 24, 48 and 72 h post inoculation (HPI), respectively; 2, 6 and 10: TDFs derived from non-inoculated resistant ICP 8863 (NIR) at 24, 48 and 72 HPI, respectively; 3, 7 and 11: TDFs derived from infected susceptible (IS) at 24, 48 and 72 HPI, respectively; 4, 8 and 12: TDFs derived from infected resistant (IR) at 24, 48 and 72 HPI, respectively. *ABCG11* ABC transporter G family member 11, *AIG1* avrRpt2 induced gene 1, *CLE24* CLAVATA3/ESR-related 24, *GAPDH* glyceraldehyde 3-phosphate dehydrogenase, *IMPORTIN* importin subunit beta-1, *MYB46* transcription factor MYB46, *SUA* SUPPRESSOR OF ABI3-5, *WOX4* WUSCHEL-related homeobox 4, *WRKY70* WRKY transcription factor 70.

Differential expressions of eight important TDFs up- and down- regulated due to *Fusarium* invasion were validated by semi-quantitative reverse transcriptase PCR (semi-qRT PCR) analysis in control and M1 infected ICP 2376 and ICP 8863 roots at three different time points after infection (24, 48 and 72 HPI) (Fig. 4, Supplementary Fig. SF6). Expression patterns of these selected TDFs were found to be consistent with the results of cDNA-AFLP analysis.

**Figure 5:**
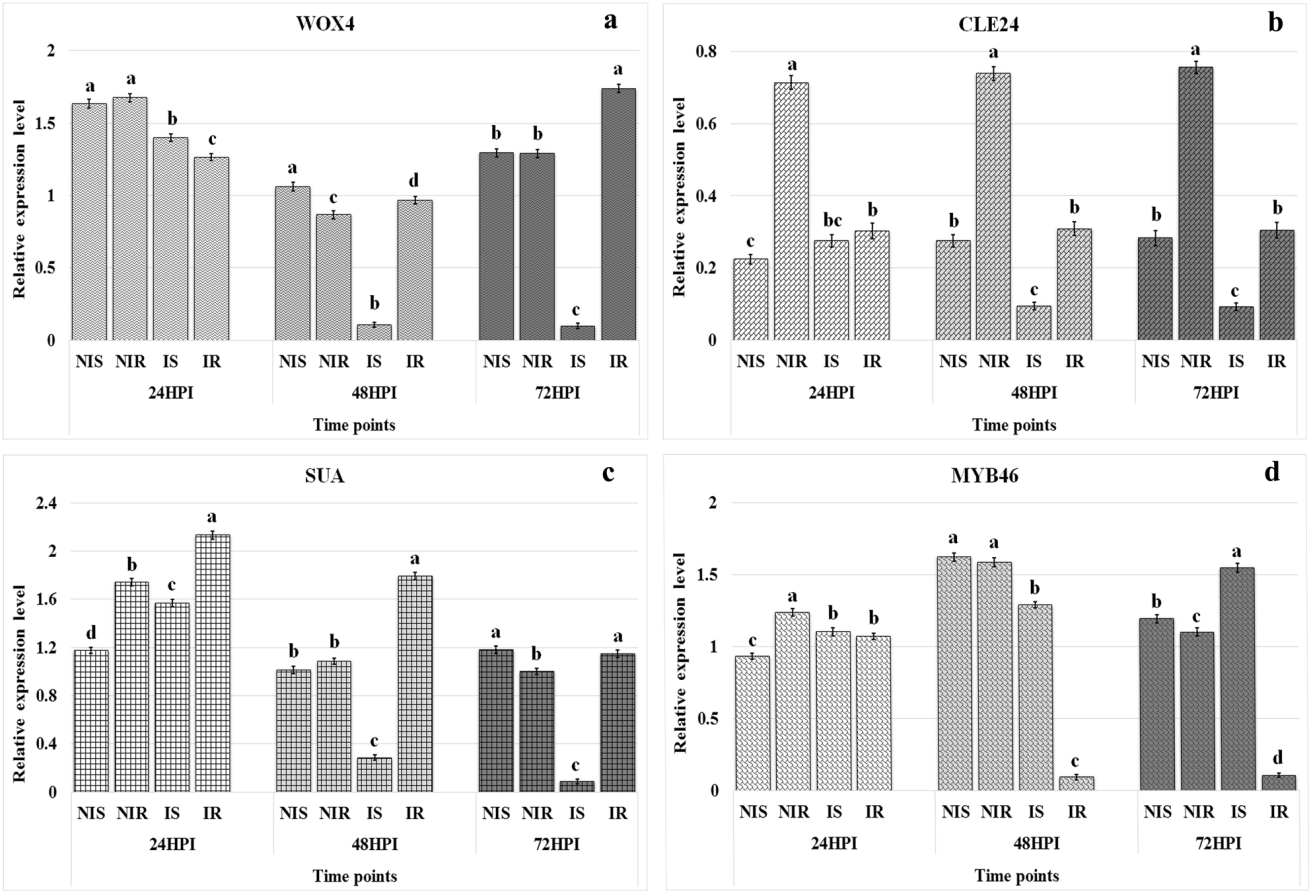
Relative expression levels of four TDFs in the control and *Fusarium udum* inoculated pigeonpea cultivars. Non-inoculated and inoculated susceptible (ICP 2376) and resistant (ICP 8863) cultivars were analyzed at three different time points by semi-quantitative reverse-transcriptase PCR. (**a**) WOX4: WUSCHEL-related homeobox 4; (**b**) CLE24: CLAVATA3/ESR-related 24; (**c**) SUA: SUPPRESSOR OF ABI3-5; (**d**) MYB46: Transcription factor MYB46. Values represent mean ± standard error from three replicates (n = 3). Letters indicate the significant differences in the means of relative expression levels for each treatment at a time point obtained using Least Significant Difference tests (p < 0.05). *HPI* hours post inoculation, *NIS* non-infected susceptible (ICP 2376), *NIR* non-infected resistant (ICP 8863), *IS* infected susceptible, *IR* infected resistant.

**Figure 6:**
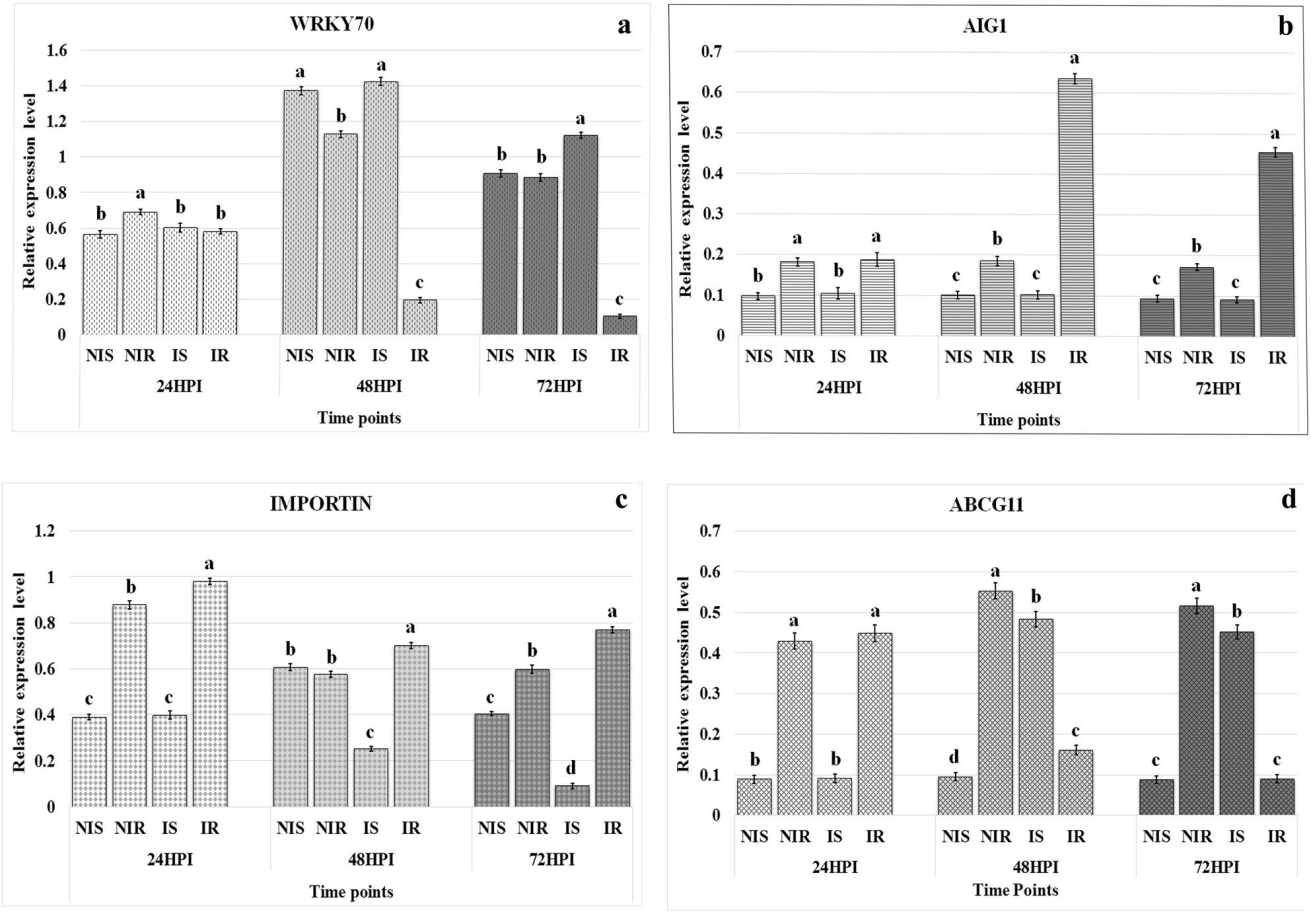
Relative expression levels of four TDFs in the control and *Fusarium udum* inoculated pigeonpea cultivars. Non-inoculated and inoculated susceptible (ICP 2376) and resistant (ICP 8863) cultivars were analyzed at three different time points by semi-quantitative reverse-transcriptase PCR. (**a**) *WRKY70* WRKY transcription factor 70; (**b**) *AIG1* avrRpt2 induced gene 1; (**c**) *IMPORTIN* importin subunit beta-1; (**d**) *ABCG11* ABC transporter G family member 11. Values represent mean ± standard error from three replicates (n = 3). Letters indicate the significant differences in the means of relative expression levels for each treatment at a time point obtained using Least Significant Difference tests (p < 0.05). *HPI* hours post inoculation, *NIS* non-infected susceptible (ICP 2376), *NIR* non-infected resistant (ICP 8863), *IS* infected susceptible, *IR* infected resistant.

TDFs PF4U18 (similar to WUSCHEL-related homeobox 4), PF18U59 (similar to CLE24 protein gene) and PF20U69 (similar to SUPPRESSOR OF ABI3-5) were down-regulated in inoculated ICP 2376 plants after 48 HPI, whereas they showed higher expression in inoculated ICP 8863 plants compared to inoculated ICP 2376 plants at 48 and 72 HPI (Fig. 5a–c). In contrast, TDF PF4D21 (similar to transcription factor MYB46) was down-regulated in infected ICP 8863 plants after 48 and 72 HPI in comparison to infected ICP 2376 plants (Fig. 5d). Simultaneously, TDF PF16D52 (similar to WRKY transcription factor 70) was also down-regulated in infected ICP 8863 plants after 48 and 72 HPI, compared to infected ICP 2376 plants (Fig. 6a). TDF PF1U1 (similar to AIG1) was up-regulated in inoculated ICP 8863 plants at 48 HPI which decreased at 72 HPI; whereas it showed no expression in inoculated ICP 2376 plants (Fig. 6b). Expression of TDF PF22U72 (similar to importin subunit beta-1) was up-regulated in infected ICP 8863 plants after 48 HPI, whereas it was downregulated in infected ICP 2376 plants at 48 and 72 HPI (Fig. 6c). In contrast, TDF PF29D92 (similar to ABC transporter G family member 11) showed decreased expressions in challenged ICP 8863 plants after 48 HPI, but the expressions was increased in infected ICP 2376 plants at 48 and 72 HPI (Fig. 6d).

## Discussion

This is the first report on transcriptomic profiling of pigeonpea–*F. udum* interaction, and identification of differential TDFs associated with defense responses in pigeonpea against *F. udum*. *F. udum* race identification has not been reported by any group, till date. However, pathogenic isolates and variants were reported in few studies^7,8^. In the previous study, present group characterized all the variants reported by Dhar et al.^7^ and some identified *F. udum* isolates from MTCC^5^. In the mentioned study, thirteen Indian *F. udum* isolates were characterized for their pathogenesis in pigeonpea^5^. Among these isolates, in M1 infected plants the different stages of disease development were prominent compared to other isolates. Hence, M1 was chosen for this study to understand early molecular responses upon infection in pigeonpea. cDNA-AFLP based comparative transcriptomic profiling of control and M1 inoculated root tissues of susceptible (ICP 2376) and resistant (ICP 8863) plants was carried out at early stage of the pathogenesis process (36 HPI), which provided evidences of early resistance/susceptibility responses in pigeonpea. The analysis of changes at transcriptional level during early response to pathogens is a key step to understand plant defence mechanisms as early induced genes are most likely to have regulatory and signaling functions which defines plant's fate after the pathogen attack^37,38^. Activation of defense responses leads to differential expression of maximum number of genes at early time points of pathogen attack^39^. Moreover, the number of fungal transcripts expressed in planta was low at early time points, may be due to low amount fungal biomass, which was comparable to previous studies on plant–fungus interactions^40^.

cDNA-AFLP analysis showed the number of down-regulated genes (151) due to pathogen induction were higher when compared to the up-regulated genes (143). Earlier studies reported similar results through cDNA-AFLP in other plant–pathogen interactions^24,40^. Forty two TDFs were functionally annotated with the help of NCBI and Uniprot databases, and thorough study of scientific literature as described in previous reports^24,30,41^. Eleven functional groups were found among 42 annotated TDFs (Supplementary Table S3). Gupta et al.^24^ and Xue et al.^22^ categorized *F. oxysporum* responsive TDFs in chickpea and common bean into 5 and 10 functional groups, respectively.

Chromosomal locations of the 48 TDFs similar to pigeonpea genes were found in NCBI and LIS databases. Pathogen induced up- and down-regulated gene fragments of different functions as well as uncharacterized TDFs were present randomly in different chromosomes. Maximum number of TDFs were present on chromosomes 11 (8) and 2 (7), which were reported marker-rich in previous studies. Singh et al.^16^ identified 4 wilt resistant candidate genes each of which were identified using nsSNPs. Two of these genes were found on chromosome 11 and other two on chromosome 2. Saxena et al.^11^ detected 3 important QTLs and candidate genomic regions present on chromosome 11. These TDFs should be of great use in the development of genetic markers for screening, identification and breeding of genotypes resistant to wilt.

Functional roles as well as possible molecular interactions of pathogen-induced TDFs were predicted based on available information in previously published works and KEGG pathway database.

### JA/SA signaling

**Figure 7:**
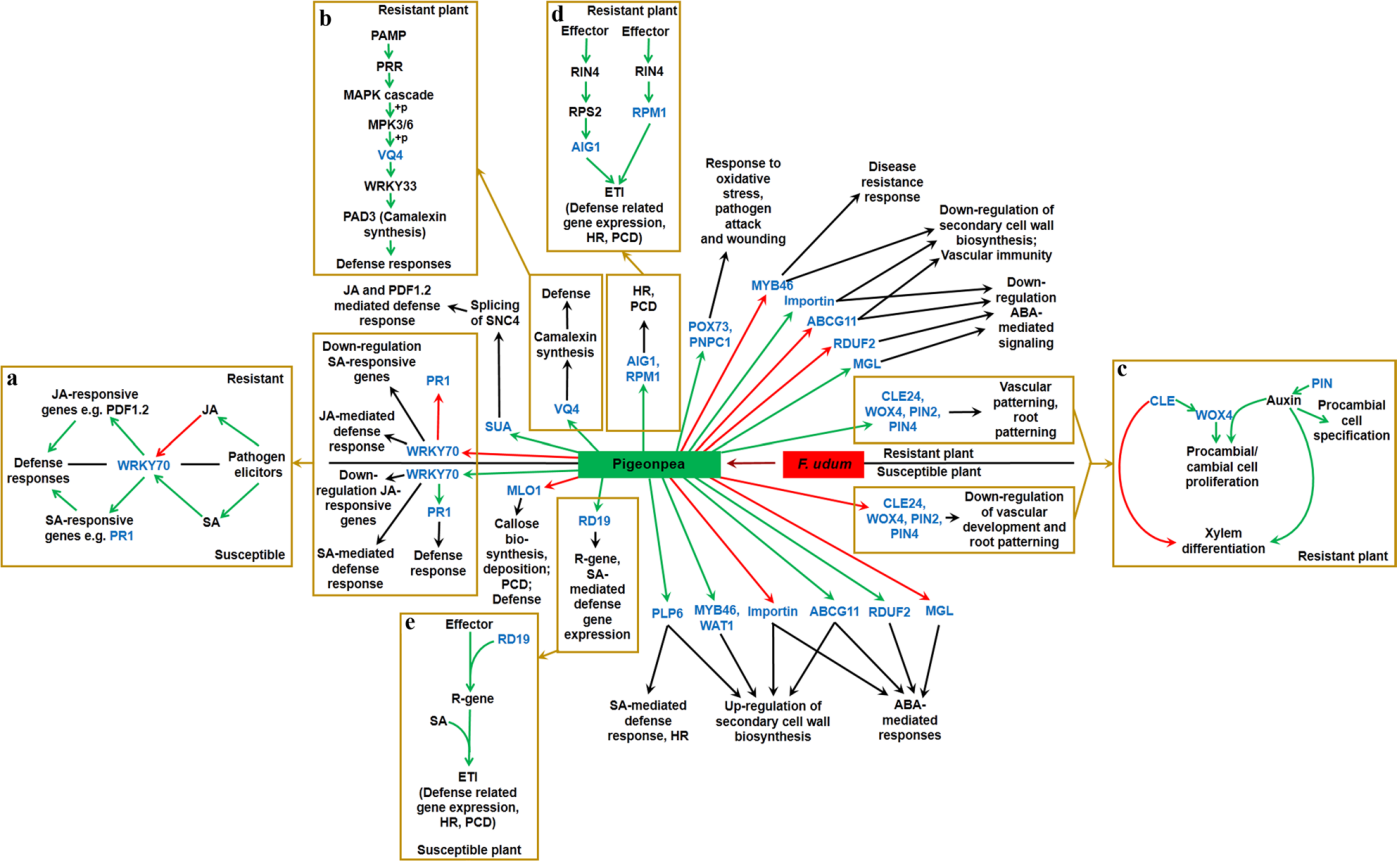
Overview of probable roles of differentially regulated TDFs during *Fusarium* wilt of pigeonpea. Green arrow: up-regulation; red arrow: down-regulation; black arrow: possible outcome due to up- or down- regulation of genes; golden arrow: elaborated pathway; blue font: genes differentially regulated in cDNA-AFLP; black font: other molecular factors with probable involvement, probable outcome of pathways. Inset (**a**): WRKY70-mediated cross-talk between Salicylic acid (SA)- and Jasmonic acid (JA)-dependent defense signaling^42^; Inset (**b**): role of VQ4 during defense signaling against pathogen^46^; Inset (**c**,**d**): regulation of RD19 (**c**^70^), AIG1 and RPM1 (**d**^67,69^) related to effector triggered immunity (ETI); Inset (**e**): Regulation of genes related to vascular development^48^. *WRKY70* WRKY transcription factor 70, *PR1* pathogenesis-related protein 1, *PDF1.2* plant defensin 1.2, *SUA* SUPPRESSOR OF ABI3-5, *SNC4* SUPPRESSOR OF NPR1-1, CONSTITUTIVE4, *VQ4* VQ motif-containing protein 4, *PAMP* pathogen-associated molecular pattern, *PRR* pattern recognition receptors, *WRKY33* WRKY transcription factor 33, *PAD3* phytoalexin deficient 3, *RPS2* resistance to *Pseudomonas syringae* protein 2, *RIN4* RPM1-interacting protein 4, *AIG1* avrRpt2 induced gene 1, *HR* hypersensitive response, *PCD* programmed cell death, *POX73* peroxidase 73, *PNPC1* cationic peroxidase 1, *MYB46* transcription factor MYB46, *ABCG11* ABC transporter G family member 11, *RDUF2* E3 ubiquitin-protein ligase RDUF2, *MGL* methionine gamma-lyase, *ABA* abscisic acid, *CLE24* CLAVATA3/ESR-related 24, *WOX4* WUSCHEL-related homeobox 4, *PIN* PIN-FORMED, *WAT1* Walls Are Thin1, *PLP6* Patatin-like protein 6, *RD19* RESPONSIVE TO DEHYDRATION19, *R-gene* resistance gene, *MLO1* MLO-like protein 1.

TDFs PF16D52 and PF14D45 similar to WRKY transcription factor 70 (WRKY70) and pathogenesis-related protein PR-1, respectively, were up-regulated in wilt-susceptible plants, whereas those transcripts were suppressed in wilt-resistant pigeonpea. WRKY70 simultaneously represses JA-responsive genes and activates SA-induced genes^42^. JA induction allows expression of several defense-response genes including *plant defensin 1.2 *(*PDF1.2*) and induce resistance response against pathogens^43^. Present study indicated that JA responsive pathway might be operational during non-compatible resistant interaction (Fig. 7). On the other hand, SA-mediated responses result in the expression of *PR1* gene^44^, which was observed in *F. udum* infected susceptible pigeonpea plants. TDF PF1U1 similar to VQ motif-containing protein 4 (VQ4, also known as MVQ1) was up-regulated in *F. udum* inoculated resistant plants. Elicitation of PAMP causes MAP kinase-mediated phosphorylation and degradation of VQ4, which allows WRKY33 to promote transcription of defense-related genes, like camalexin biosynthetic genes^45,46^. In the present interaction this might be an important pathway to get early defense responses in resistant pigeonpea.

### Regulation of SUPPRESSOR OF ABI3-5

TDF PF20U69 similar to SUPPRESSOR OF ABI3-5 (SUA) was up-regulated in inoculated resistant pigeonpea plants. Splicing of *CHITIN ELICITOR RECEPTOR KINASE 1* (*CERK1*) and *SUPPRESSOR OF NPR1-1*, *CONSTITUTIVE4* (*SNC4*), is regulated by SUA. CERK1 and SNC4 encodes for receptor-like kinases which contributes to defense responses in plants. Recently, aberrant splicing of *SNC4* and *CERK1* was found in the *sua* knock-out mutant of *Arabidopsis* which showed susceptibility to *Pseudomonas syringae* infection^47^. Over-expression of this gene indicated resistance response in pigeonpea.

### Alteration of vascular patterning genes

TDFs PF18U59 and PF4U18, similar to CLAVATA3/ESR-related 24 (CLE24) and WUSCHEL-related homeobox 4 (WOX4), respectively, were up-regulated in inoculated resistant plants. CLAVATA3/ESR stimulates procambial/cambial cell proliferation and specifically inhibits xylem differentiation. One direct outcome of this signal transduction is up-regulation of *WOX4* transcription factor. *WOX4* is specifically expressed in the procambium/cambium stem cell niche where it functions to stimulate cell proliferation. The combination of both auxin and CLAVATA3/ESR signaling provides the specific conditions to promote cambial stem cell proliferation in the shoot apical meristem (SAM). WOX4 mediates the auxin dependent induction of cambium activity^48,49^. PF14U44 similar to auxin efflux carrier component 2 (PIN2) and PF11U40 similar to auxin efflux carrier component 4 (PIN4) was up-regulated in resistant plants. PIN are auxin efflux protein localized at plasma membrane expressed during very early stages of procambial cell specification. Procambial and cambial cell proliferation, and xylem differentiation are positively regulated by PIN. PIN2 may contribute to root-specific auxin transport, and mediate gravitropism of root. Its particular localization suggests a role in the translocation of auxin towards the elongation zone. PIN4 maintains the endogenous auxin gradient, which is essential for correct root patterning^48,50^.

Up-regulation of these genes in infected resistant pigeonpea cultivars indicated reprogramming of the vascular gene regulatory networks towards controlled differentiation of SAM and procambium to overcome *F. udum* induced anomalous signal in xylem cells.

### Regulation of genes specific to secondary cell wall biosynthesis

TDF PF22U72 similar to importin subunit beta-1 was up- and down-regulated in wilt-resistant and -susceptible pigeonpea plants, respectively. *Arabidopsis* importin beta -domain family proteins are important negative effectors of ABA mediated drought tolerance. *SAD2* encodes for an importin beta -domain family protein. SAD2 was needed for nuclear import of MYB4, which is involved in repression of secondary cell wall biosynthesis genes^48,51^. TDF PF4D21 similar to transcription factor MYB46-like was down-regulated in resistant cultivars. *MYB46* transcription factor is required to trigger expression of several transcription factors, which contributes to formation of secondary cell wall. Increased resistance to *Botrytis cinerea* was observed in the *myb46* knock-down mutant *Arabidopsis* plants. This resistance was linked to an early down-regulation of cellulose synthase (*CesA*) genes following *B. cinerea* infection. Down-regulation of *MYB46* or *CesA* resulted in activation of JA-mediated defense response^52,53^. TDF PF10D39 having homology with Walls Are Thin1 (WAT1)-related protein At4g28040-like was down-regulated in wilt-resistant pigeonpea plants. Resistance to vascular pathogens was achieved in *Arabidopsis* by the inactivation of *WAT1* gene, also involved in secondary cell-wall deposition. SA also plays important role in this resistance^54^. TDF PF29D92 similar to ABC transporter G family member 11 was up- and down-regulated in wilt-susceptible and -resistant plants, respectively. *AtABCG11* is induced by ABA and wound stress. *AtABCG11* contributes to cutin and wax secretion on the leaf epidermis. *AtABCG11* may also be related to suberin formation in roots and lignifications^55,56^. ABCG11 is likely to be involved in patterning of the vascular system^57^.

The evidences of down-regulation of these genes indicated that *Fusarium* wilt-resistance may not be conferred by remodeling the cell wall as a passive barrier, but by the activation of immune responses, mostly localized in the vascular system, which is recognized as vascular immunity^52,54,58^. Alternately, up-regulation of these genes in susceptible pigeonpea cultivar had the possible roles in excessive vascular cell division as well as repair and regeneration of secondary cell wall during *F. udum* infection.

### Regulation of patatin-like protein 6

PF4D22 similar to patatin-like protein 6 (PLP6, also known as pPLAIIIα or AtPLAIIβ) was up-regulated in inoculated susceptible plants. PLP6 hydrolyzes phospholipids as well as galactolipids^59^. *Oryza sativa* PLP6 transcript level increased under drought conditions^60^. *Petunia* plants over-expressing *A. thaliana* PLP6 showed rapid and intense hypersensitive response (HR) during *B. cinerea* and *P. syringae* infection. In these plants enhanced expression of SA-mediated pathway was observed; but JA-dependent PR gene expression was not increased^61^. PLP6 over-expressing *O. sativa* plants exhibited enhanced expression of *CesA* genes important for primary and secondary cell wall formation^62^.

### ABA mediated signaling

AtRDUF2 (similar to TDF PF3D15) positively regulates responses in *Arabidopsis* during drought stress in an ABA-dependent manner^63^. *AtABCG11* (TDF PF29D92), as mentioned earlier, was also induced by ABA. PF1D6 similar to vesicle-associated protein 2-2 (VAP2-2, also known as VAP27-2), which plays a role in vesicular trafficking, was up-regulated in maize during drought stress in ABA dependent manner^64^. These three TDFs were up-regulated in wilt-susceptible plants in comparison to resistant counterpart. On the other hand, Methionine gamma-lyase (MGL), similar to TDF PF19U66, activity is highly increased during drought stress and it is negatively regulated by ABA^65,66^. *Arabidopsis* Importin beta -domain family proteins (subunit of such a protein found similar to TDF PF22U72) are important negative effectors of ABA mediated drought tolerance. These two TDFs (PF19U66 and PF22U72) were up-regulated in wilt-resistant plants in comparison to susceptible counterpart. Altogether, it may be inferred that ABA mediated signaling was down-regulated during resistance responses in pigeonpea wilt.

### ETI responses

TDFs PF1U2 similar to protein AIG1 (avrRpt2 induced gene) and PF25U81 similar to disease resistance protein RPM1 were up-regulated in inoculated resistant ICP 8863 plants. NB-LRR R proteins, RPM1 and RPS2 detect changes in RPM1-interacting protein 4 (RIN4) triggered by AvrRpm1 and AvrRpt2 effectors, respectively and lead to ETI. Immediately after pathogen infection, AIGl is induced by avrRpt2 and RPS2, and may play role in inducing cell death^67–69^. TDF PF3D16 similar to cysteine protease RESPONSIVE TO DEHYDRATION19A (RD19A) was up-regulated in inoculated susceptible plants. A nuclear complex is formed by association of RD19 and *R. solanacearum* type III effector, which activates *R. solanacearum* specific R-gene to initiate defense response^70^ (Fig. 7). RD19A in *Arabidopsis* was induced by water deficit, salt stress and aphids^71,72^.

### ROS mediated signaling

TDFs PF10U37 similar to *peroxidase 73* (POX73) and PF32U99 similar to *cationic peroxidase 1* (PNPC1) were up-regulated in resistant plants. Peroxidases contributes to cross-linking of cell wall components, lignin and suberin formation, auxin metabolism, phytoalexin synthesis, and the metabolism of reactive nitrogen species (RNS) and ROS. They also contribute to symbiosis, normal cell growth, wound healing and defense response during biotic stress^73,74^.

Several other TDFs with varied functions were up- and down-regulated in inoculated resistant and susceptible plants (Table 1). Collectively, functional study of TDFs indicated different key events, which could play crucial role in defense responses of the *F. udum* inoculated resistant plants. Pathogen invasion prominently triggered JA mediated defense responses in wilt resistant cultivar of pigeonpea, whereas SA mediated pathway was up-regulated in the susceptible counterpart. It seems that, instead of up-regulation of cell wall remodeling genes, the downstream activation of vascular immunity could play an important role in resistance. It can be assumed that auxin mediated induction of different vascular developmental genes played their roles in reprogramming of the vascular network towards controlled differentiation of meristem to overcome *F. udum* induced tissue anomaly. Interestingly, abscisic acid mediated responses were up-regulated in wilt susceptible pigeonpea plants and down-regulated in resistant plants.

This is the first report on identification and characterization of transcripts and prediction of molecular pathways with possible roles during wilt development. This study has provided valuable insights on molecular basis of pigeonpea–*Fusarium* interactions and will be the basis for characterization of concerned genes through functional genomics approaches. The potential of the identified genes will be validated through the analysis of disruption or deletion mutants. Advanced techniques such as virus-induced gene silencing, T-DNA mutagenesis and CRISPR mediated gene editing approaches will be used for such mutant generation. Functional identification of those validated genes will be helpful in development of wilt resistant pigeonpea plants through genomics-assisted breeding, genetic engineering or genome editing techniques. Additionally, identification of wilt resistance or susceptibility in pigeonpea will be helpful to design appropriate phytosanitary measures for the pathogen removal to overcome quarantine barriers related to trade.

## Materials and methods

### Pigeonpea cultivars

Seed of wilt-susceptible ICP 2376 and wilt-resistant ICP 8863 cultivars of pigeonpea were acquired from International Crops Research Institute for the Semi-Arid Tropics (Patancheru, Andhra Pradesh, India). Seeds were sown in soilrite mix (Keltech Energies Ltd., Bangalore, India) and grown at 22–25 °C and 14–16 h photoperiod. The humidity was maintained at 35–40%.

### Isolate of *F. udum*

M1, an isolate of *F. udum*, was acquired from Microbial Type Culture Collection (Institute of Microbial Technology, Chandigarh, India). Single colony of M1 was inoculated on potato dextrose agar medium (PDA) and allowed to grow in dark at 25 °C for 8 days.

### Inoculation of pigeonpea with M1

ICP 2376 and ICP 8863 seeds were surface sterilized by HgCl_2_ (0.05%), sown on soilrite, and grown under the above mentioned conditions. Twelve-fourteen days old Seedlings were inoculated with M1 using the method outlined by Purohit et al.^5^. Conidia suspension of 1 × 10^6^ ml^−1^ concentration was obtained from 12–14 days old M1 culture on PDA and 50 ml of suspension was mixed with 200 g of sand:chickpea meal (9:1), and kept at 25 °C under dark for 12–14 days. *Fusarium* infested mix was incorporated with 2 kg of sand:soilrite (1:1). Seedlings were transferred to infested sand–soilrite mixture and one seedling was grown per pot (9 cm height, 7 cm diameter). Plants grown in non-inoculated mix were used as control. Control and inoculated plants of both cultivars were grown in mentioned conditions with adequate watering. Changes in root- and shoot-morphology were studied in control and infected plants of both the cultivars till 8 DPI. Anatomical study of non-infected and infected roots of both cultivars was performed by staining transverse sections with trypan blue–lactophenol at 12-h intervals till 8 DPI. Each experiment was repeated three times.

### Extraction of RNA and preparation of double-stranded cDNA

Control and M1 infected roots of both cultivars (ICP 2376 and ICP 8863) were collected at 36 HPI for RNA preparation. Three plants (biological replications) of each control and treatment were used for analysis. Isolation of total RNA was done from 500 mg frozen roots of each samples using Trizol reagent (Ambion) according to the protocol outlined by the manufacturer. Thoroughly washed root samples were ground to a fine powder with liquid nitrogen using a pestle and mortar. Trizol reagent (5 ml) was added to the samples and ground again to make slurry. Then 1 ml of chloroform was added, transferred to centrifuge tubes and thoroughly mixed by inverting. After centrifugation at 10,000*g* for 30 min at 4 °C, aqueous phase was taken from the mixture and ice-cold isopropanol (equal volume of the aqueous phase) was added, distributed in microfuge tubes, mixed by inverting, kept at 4 °C for 45 min and centrifuged at 15,000*g* for 30 min at 4 °C. Pellet was washed with ice cold 70% ethanol by centrifugation at 15,000*g* for 15 min at 4 °C. After removing 70% ethanol, pellets were dried in laminar flow, dissolved in RNase free water and stored in -80 °C. Total RNA was checked for integrity and quality in a 1.2% formamide denaturing gel run in 3-*N*-morpholino propane sulphonic acid (MOPS) buffer. Quantity of total RNA was determined using a spectrophotometer. Qiagen Oligotex mRNA minikit (Qiagen, Hilden, Germany) was used to purify mRNA from total RNA. Double-stranded cDNA was prepared from each poly A mRNA (500 ng) sample using SMARTer PCR-cDNA synthesis kit (Clontech Laboratories, Inc., Dalian, China) following manufacturer’s instructions.

### cDNA-AFLP profiling

Double-stranded cDNA (250 ng) samples were used for AFLP reactions as demonstrated in our previous work^5^. cDNA samples were digested by *Eco*RI and *Mse*I, followed by ligation of *Eco*RI (5′-CTCGTAGACTGCGTACC-3′ and 3′-CTGACGCATGGTTAA-5′) and *Mse*I (5′-GACGATGAGTCCTGAG-3′ and 3′-TACTCAGGACTCAT-5′) adapters. *Eco*RI + A primer (E-A, 5′-GACTGCGTACCAATTCA-3′) and *Mse*I + 0 primer (M-0, 5′-GATGAGTCCTGAGTAA-3′) were used for pre-amplification of adapter-ligated products. Pre-amplified products were taken in equal amount for selective amplification with *Mse*I primers (M-GA, M-GT, M-TG, M-CA, M-CG, M-TC, M-CAC, M-CAG, M-CAT, M-CTA, M-CTG) and *EcoR*I primers (IRDye 700 labelled E-ACA, E-AA, E-AG, IRDye 800 labelled E-AAG, E-ACT, E-AT) (Supplementary Table S1). Separation of the labelled selectively amplified products on 6.5% urea-PAGE was scanned with the LI-COR 4300 DNA Analyzer and visualized on the attached computer monitor. The gel images were examined and the primer combinations were checked for quality of the banding profile; number, resolution and size of the polymorphic bands produced due to infection. Forty eight out of the 66 primer combinations were selected for the final cDNA-AFLP profiling. All the double stranded cDNA samples were again subjected to AFLP profiling using the selected 48 selective amplification primer combinations (Supplementary Table S2) and the selectively amplified products were separated on urea-PAGE (6.5%). Gels were placed on a 3 MM Whatmann paper, marked at each corner, wrapped in a serene wrap and dried on a gel dryer (GeNei, Bangalore, India) at 55 °C. Marked dried gels were photographed in ODYSSEY Infrared Imager (LI-COR Biosciences).

### Isolation, re-amplification, cloning and sequencing of transcript-derived fragments

Printed and marked gel images were aligned with the dried gels and differentially expressed pathogen induced bands were excised carefully from gel using a sharp sterile scalpel avoiding any sort of contamination. Excised gels were viewed and photographed again in ODYSSEY Infrared Imager to check for correct excision of the pathogen induced differential bands. Each excised gel band containing TDF was stored in 50 μl sterile water overnight, vortexed, centrifuged and the supernatant was collected. Isolated TDFs were re-amplified using corresponding primers in a reaction mix of 15 μl using the amplification program: 2 min at 94 °C; 35 cycles of 30 s at 94 °C, 56 °C for 30 s, and 72 °C for 30 s; and 5 min at 72 °C. Re-amplification products were then run in 2% agarose gel, followed by visualization using UV transilluminator (UVP, LMS-20). Re-amplified PCR products were extracted (Qiagen, Hilden, Germany) using the protocol outlined by manufacturer and dissolved in 15 μl HPLC grade water.

Purified TDFs were then incorporated in pGEM-T Easy Vector System I (Promega Corp., Madison, WI) using the protocol outlined by the manufacturer. The sequencing reactions of the cloned TDFs were carried out on ABI 3730xl DNA analyzer (Applied Biosystems, Foster city, CA, U.S.A.) using T7 and SP6 promoter primers, and Big dye terminator V3.1 cycle sequencing Kit (Applied Biosystems).

### Analyses of sequences using bioinformatic tools

TDF sequences were analyzed using Blastn algorithm (http://blast.ncbi.nlm.nih.gov/Blast.cgi) to find out their homology with known nucleotide sequences in NCBI non redundant GenBank nucleotide database^32^. Homology of TDF sequences were also searched in the LIS and Phytozome databases using Blastn algorithm (https://legumeinfo.org/blast/nucleotide/nucleotide and https://phytozome.jgi.doe.gov/pz/portal.html#!search?show=BLAST in LIS and Phytozome, respectively)^33,34^. Each TDF was functionally annotated by analyzing previous studies and taking information from the Uniprot website (https://www.uniprot.org)^35^. The TDFs were named with initials ‘PF’ (Pigeonpea-Fusarium) and each of them was characterized for expression profile, sequence length, homology (NCBI hit, LIS target name), function, query cover, E-value, identity and genomic location by chromosome number. Sequences of the TDFs were submitted to NCBI. Probable functions and molecular interactions among these TDFs were predicted based on analysis of the scientific literature and the information reported by the KEGG PATHWAY database (https://www.genome.jp/kegg/pathway.html)^36^.

### Analysis of TDFs by semi-quantitative reverse transcriptase PCR

RNA was extracted from Control and M1 infected ICP 2376 and ICP 8863 roots at 24, 48 and 72 HPI. First-strand cDNA was synthesized from each RNA (5 μg) samples with Oligo(dT)18 primers and RevertAid Reverse Transcriptase (Thermo Fisher Scientific) following protocol outlined by the manufacturer. Eight TDFs were selected and TDF specific primers were designed (Supplementary Table S5). PCR was carried out in 15 μl reaction mix using each cDNA (100 ng) sample following the mentioned program: 95 °C for 2 min; 35 cycles of 95 °C for 30 s, 49–54 °C for 30 s and 72 °C for 30 s; 72 °C for 8 min. Agarose gel electrophoresis (2%) was run to analyze the band intensities using ImageJ software^75^. Relative intensities of the genes were analyzed using glyceraldehyde 3-phosphate dehydrogenase (GAPDH) as reference gene. Each reaction was repeated three times, standard error was calculated for each gene. In the figures, values were represented as mean ± standard error. Statistical analysis of all data was carried out using Statistica Software V. 10.0^76^. Mean values of relative expression level (n = 3) of control and infected, susceptible and resistant pigeonpea cultivars at every time point were compared using one way analysis of variance (ANOVA) and mean differences were analyzed using Least Significant Difference (LSD) post-hoc tests (p < 0.05). In the figures, different letters indicate the significant differences in the means of relative expression levels for each treatment at every time point.

### Ethical approval

All studies on plants complied with relevant institutional, national, and international guidelines and legislation.

### Supplementary Information

Supplementary Information.
