# Author Correction: Comparative transcriptomic profiling of susceptible and resistant cultivars of pigeonpea demonstrates early molecular responses during *Fusarium udum* infection

**DOI:** 10.1038/s41598-023-48314-y

**Published:** 2023-11-30

**Authors:** Arnab Purohit, Sanatan Ghosh, Shreeparna Ganguly, Madan Singh Negi, Shashi Bhushan Tripathi, Rituparna Kundu Chaudhuri, Dipankar Chakraborti

**Affiliations:** 1 Department of Biotechnology, St. Xavier’s College (Autonomous), 30, Mother Teresa Sarani, Kolkata, West Bengal 700016 India; 2 Department of Genetics, University of Calcutta, 35, Ballygunge Circular Road, Kolkata, 700019 India; 3 Sustainable Agriculture Division, TERI, India Habitat Center Complex, Lodhi Road, New Delhi, 110003 India; 4 TERI-School of Advanced Studies, 10, Institutional Area, Vasant Kunj, New Delhi, 110070 India; 5 Department of Botany, Krishnagar Govt. College, Krishnagar, West Bengal 741101 India

Correction to: *Scientific Reports* https://doi.org/10.1038/s41598-021-01587-7, published online 16 November 2021

The original version of this Article contained an error in Figure 1. Due to a mistake in figure assembly, the panel “ICP 2376, noninfected, vascular bundle” was partially overlapping with a panel of Figure 7 in^1^. The original Figure 1 and accompanying legend appear below.

**Figure 1:**
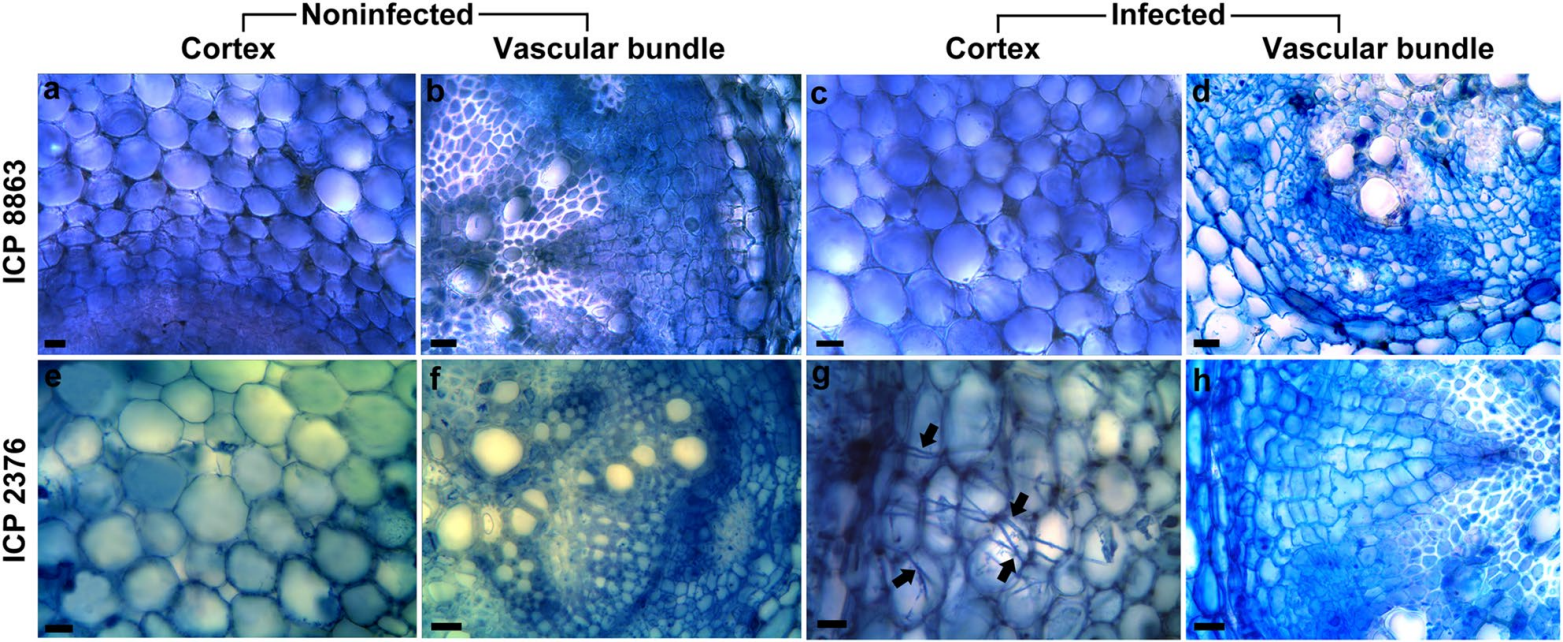
Transverse sections of control and *Fusarium udum* isolate M1 inoculated roots of pigeonpea at 36 h post inoculation. (**a**,**b**) Non-inoculated control roots of resistant ICP 8863 cultivar, (**c**,**d**) roots of ICP 8863 inoculated with M1, (**e**,**f**) non-inoculated control roots of the susceptible ICP 2376 cultivar, (**g**,**h**) roots of ICP 2376 inoculated with M1. Arrows indicate fungal mycelia. Bars represent 10 μm.

The original Article has been corrected.
