# Supplementary material for: Comparative transcriptomic profiling of susceptible and resistant cultivars of pigeonpea demonstrates early molecular responses during Fusarium udum infection
Source: Sci Rep. 2021 Nov 16;11:22319. doi: 10.1038/s41598-021-01587-7 (PMC8595609; doi:10.1038/s41598-021-01587-7)
Supplement: Supplementary file 1 — Supplementary Information. [file 41598_2021_1587_MOESM1_ESM.pdf]

**Comparative transcriptomic profiling of susceptible and resistant cultivars of pigeonpea demonstrates early molecular responses during *Fusarium udum* infection**

Arnab Purohit[1,2], Sanatan Ghosh[2], Shreeparna Ganguly[1], Madan Singh Negi[3], Shashi Bhushan Tripathi[4], Rituparna Kundu Chaudhuri[5], Dipankar Chakraborti[2*]

[1] Department of Biotechnology, St. Xavier's College (Autonomous), 30, Mother Teresa Sarani, Kolkata, West Bengal, 700016, India

[2] Department of Genetics, University of Calcutta, 35, Ballygunge Circular Road, Kolkata, 700019, India

[3] Sustainable Agriculture Division, TERI, India Habitat Center Complex, Lodhi Road, New Delhi, 110003, India

[4] TERI-School of Advanced Studies, 10, Institutional Area, Vasant Kunj, New Delhi, 110070, India

[5] Department of Botany, Krishnagar Govt. College, Krishnagar, West Bengal, 741101, India

* Dipankar Chakraborti
dcgntcs@caluniv.ac.in

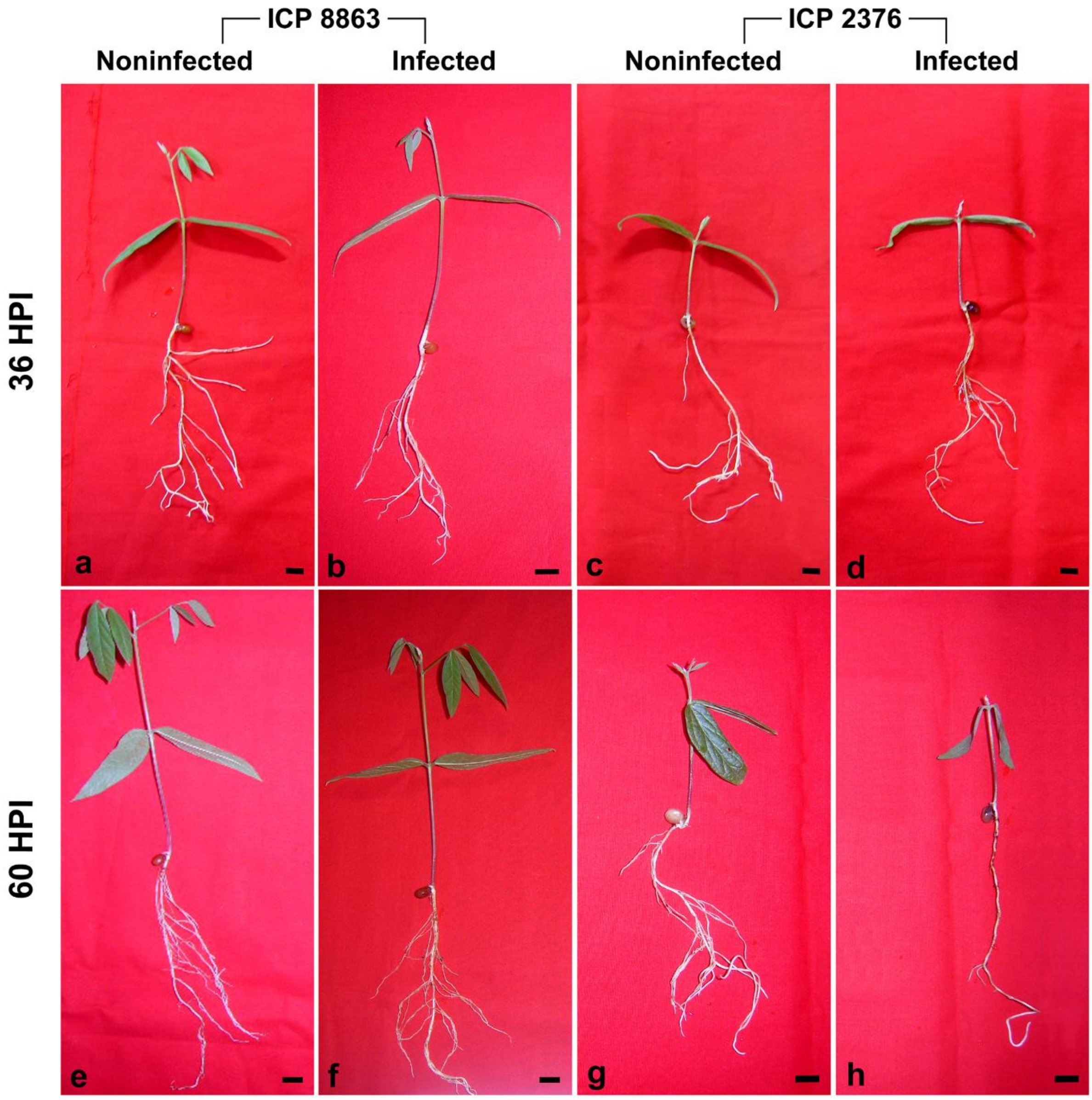


**Supplementary Fig. SF1.** Seedlings of pigeonpea at 36 (a-d) and 60 (e-h) hours post inoculation (hpi) with *Fusarium udum* isolate M1. (a, e) Non-inoculated control seedlings of wilt resistant ICP 8863 cultivar, (b, f) ICP 8863 seedlings inoculated with M1, (c, g) Non-inoculated control seedlings of wilt susceptible ICP 2376 cultivar and (d, h) ICP 2376 seedlings inoculated with M1. Bar represents 1 cm.

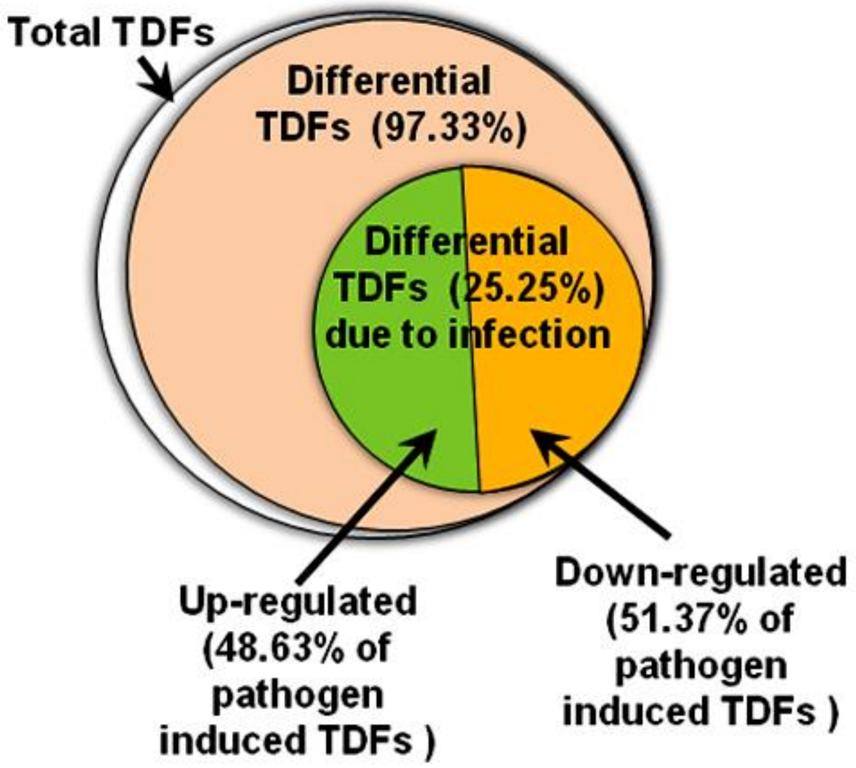


**Supplementary Fig. SF2.** Percentages of transcript-derived fragments (TDFs) generated during cDNA-AFLP analysis.

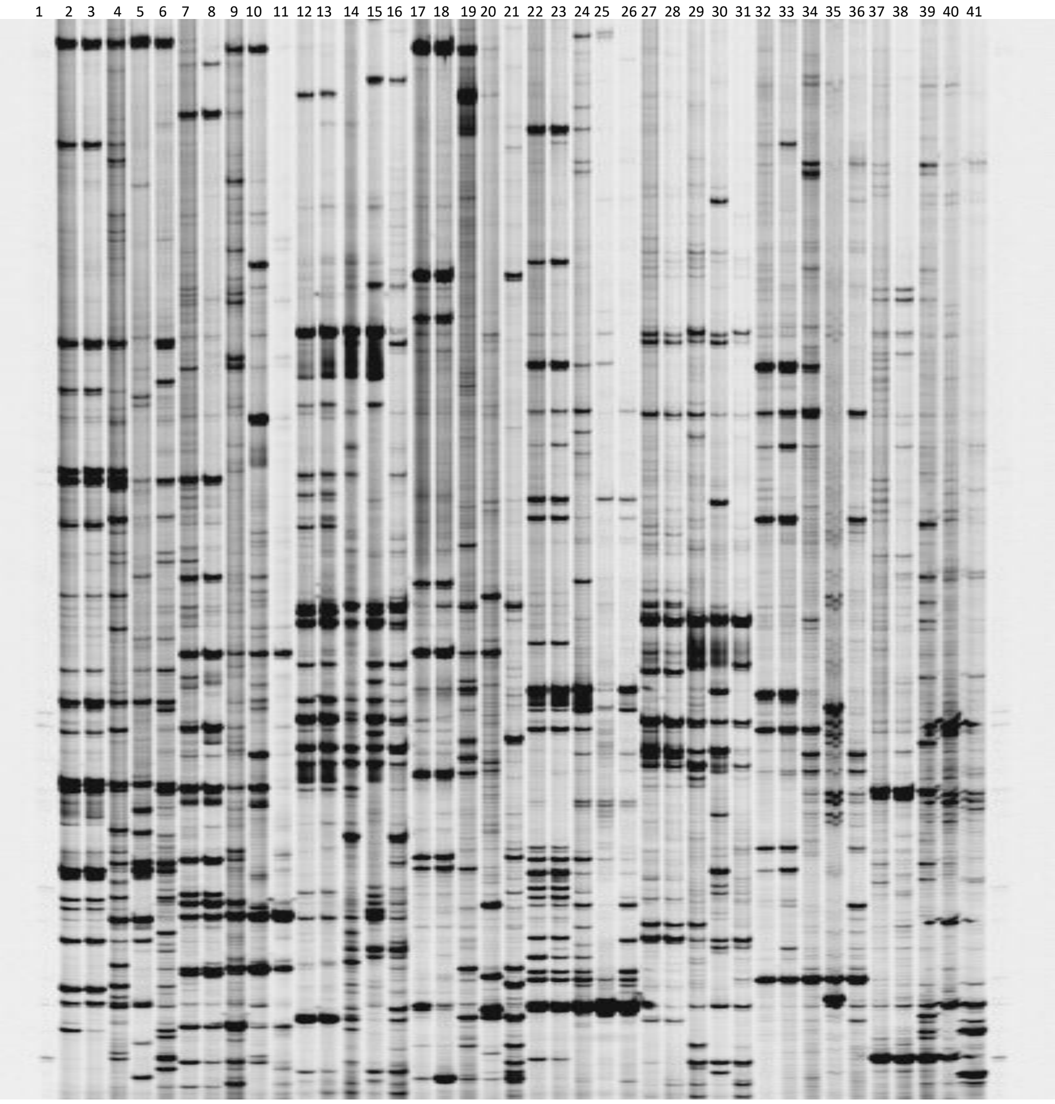


**Supplementary Fig. SF3.** Representative cDNA-AFLP profiles of non-inoculated and *Fusarium udum* inoculated ICP 2376 and ICP 8863 samples using different primer combinations. Lanes 3,8,13,18,23,28,33,38: non-inoculated control ICP 2376; lanes 4,9,14,19,24,29,34,39: non-inoculated control ICP 8863; lanes 5,10,15,20,25,30,35,40: inoculated ICP 2376; lanes 6,11,16,21,26,31,36,41: inoculated ICP 8863; The primer combinations used were; lanes 3 to 6: E-AT/M-GA; lanes 8 to 11: E-AT/M-GT; lanes 13 to 16: E-AT/M-TG; lane 18 to 21: E-AT/M-TC; lane 23 to 26: E-AT/M-CA; lanes 28 to 31: E-AT/M-CG; lanes 33 to 36: E-AT/M-CAT; lanes 38 to 41: E-AT/M-CTA. Lane 1: 50 to 1500 bp size standard (LI-COR Biosciences).

a

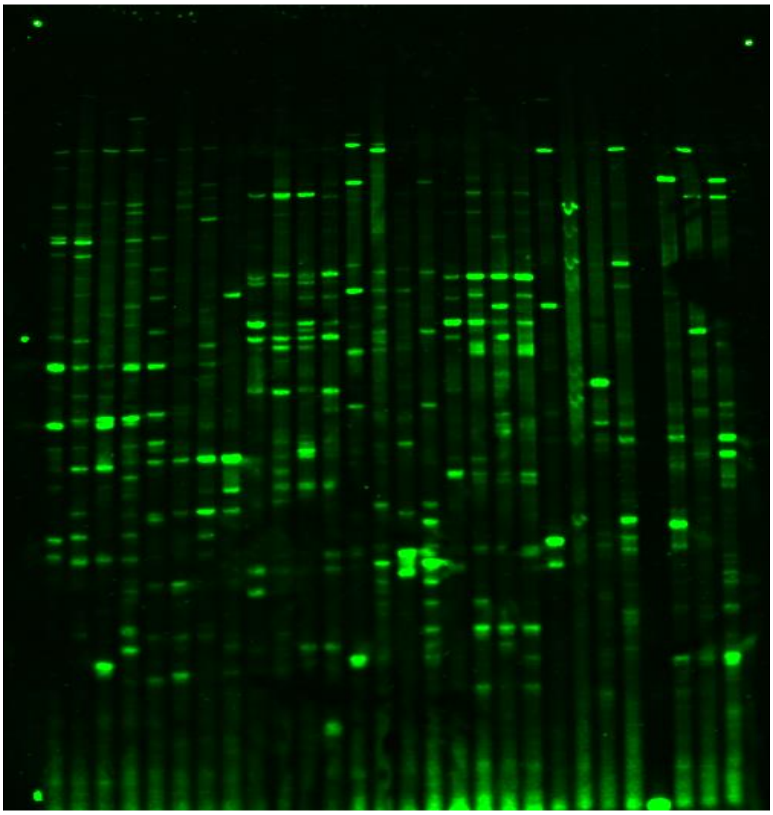
1 2 3 4 5 6 7 8 9 10 11 12 13 14 15 16 17 18 19 20 21 22 23 24 25 26 27 28

b

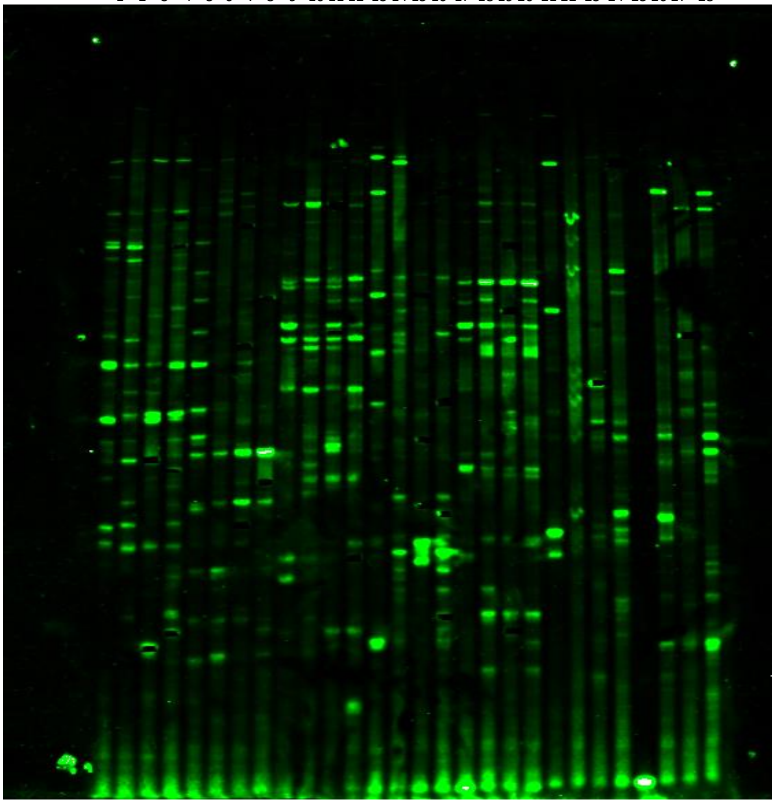
1 2 3 4 5 6 7 8 9 10 11 12 13 14 15 16 17 18 19 20 21 22 23 24 25 26 27 28

**Supplementary Fig. SF4.** Representative cDNA-AFLP profiles of non-inoculated and *Fusarium udum* inoculated ICP 2376 and ICP 8863 samples using different primer combinations, photographed (a) before and (b) after excision of TDFs from the gel. Lanes 1,5,9,13,17,21: non-inoculated control ICP 2376; lanes 2,6,10,14,18,22: non-inoculated control ICP 8863; lanes 3,7,11,15,19,23: inoculated ICP 2376; lanes 4,8,12,16,20,24: inoculated ICP 8863; The primer combinations used were; lanes 1 to 4: E-AT/M-GA; lanes 5 to 8: E-AT/M-GT; lanes 9 to 12: E-AT/M-TG; lane 13 to 16: E-AT/M-TC; lane 17 to 20: E-AT/M-CG; lanes 21 to 24: E-ACT/M-GA. (a) Before and (b) after excision.

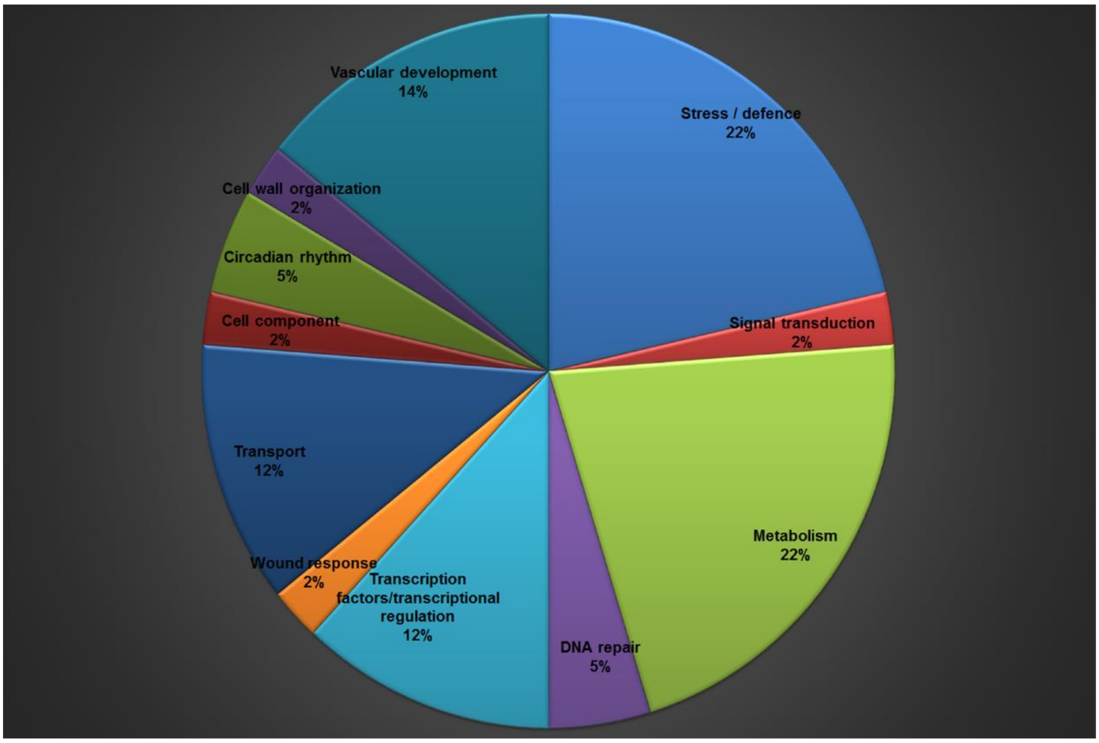


**Supplementary Fig. SF5.** Different types of transcript-derived fragments obtained due to *Fusarium udum* attack.

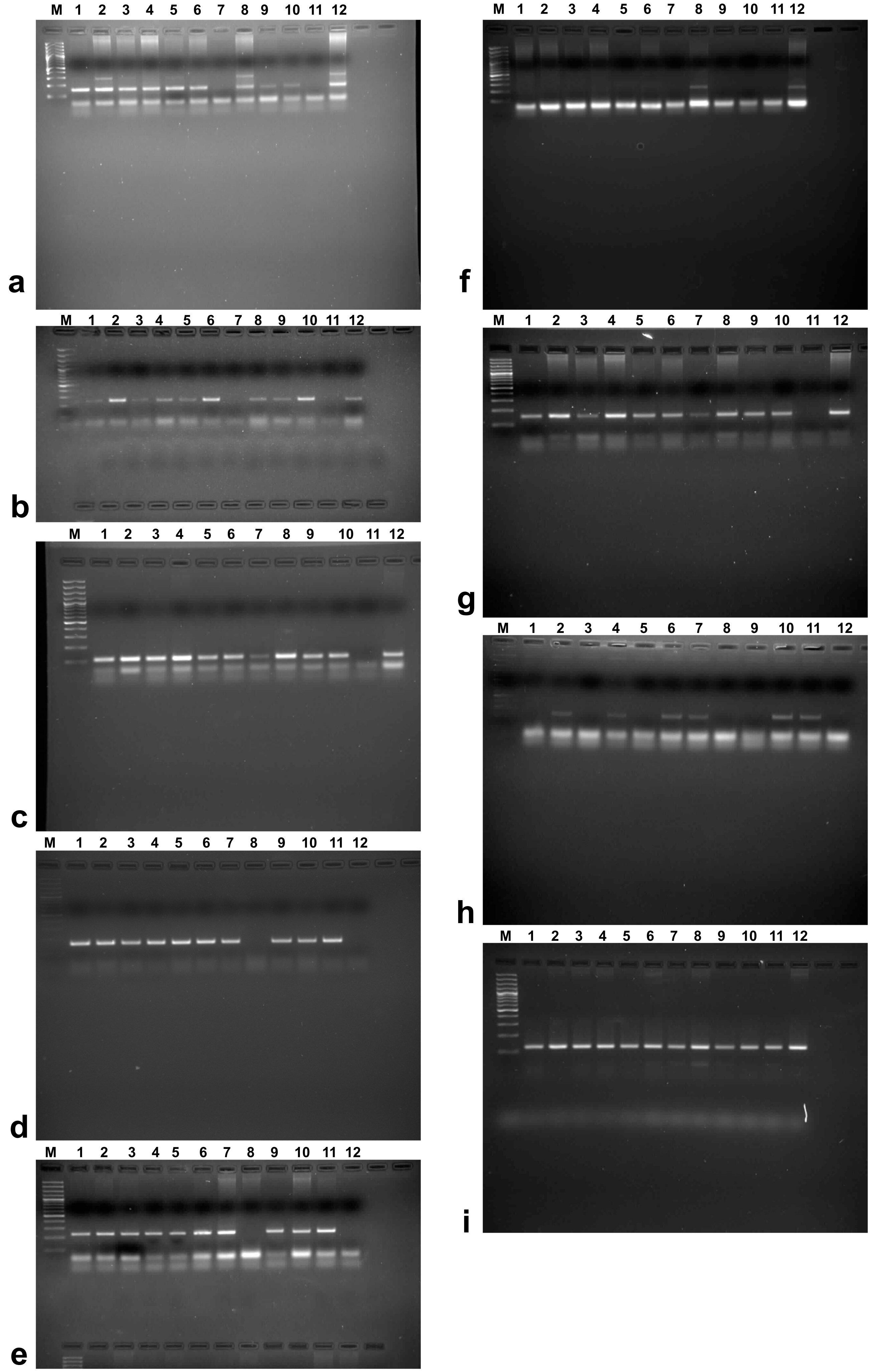
M 1 2 3 4 5 6 7 8 9 10 11 12
a
b
c
d
e
f
g
h
i

**Supplementary Fig. SF6.** Semi-quantitative reverse-transcriptase PCR amplification patterns of selected genes in the control and *Fusarium udum* inoculated susceptible ICP 2376 and resistant ICP 8863 pigeonpea cultivars at three different time points. Lane M, molecular weight marker, Lanes 1, 5 and 9: TDFs derived from non-inoculated susceptible ICP 2376 (NIS) at 24, 48 and 72 hours post inoculation (HPI), respectively; 2, 6 and 10: TDFs derived from non-inoculated resistant ICP 8863 (NIR) at 24, 48 and 72 HPI, respectively; 3, 7 and 11: TDFs derived from infected susceptible (IS) at 24, 48 and 72 HPI, respectively; 4, 8 and 12: TDFs derived from infected resistant (IR) at 24, 48 and 72 HPI, respectively. (a) WOX4: WUSCHEL-related homeobox 4; (b) CLE24: CLAVATA3/ESR-related 24; (c) SUA: SUPPRESSOR OF ABI3-5; (d) MYB46: Transcription factor MYB46; (e) WRKY70: WRKY transcription factor 70; (f) AIG1: avrRpt2 induced gene 1; (g) IMPORTIN: Importin subunit beta-1; (h) ABCG11: ABC transporter G family member 11; (i) GAPDH: Glyceraldehyde 3-phosphate dehydrogenase.

**Supplementary Table S1** Selective amplification primers

| Primers | Sequences (5’-3’) |
|---|---|
| E-AA | 5′-GACTGCGTACCAATTCAA-3′ |
| E-AG | 5′-GACTGCGTACCAATTCAG-3′ |
| E-ACA | 5′-GACTGCGTACCAATTCACA-3′ |
| E-AAG | 5′-GACTGCGTACCAATTCAAG-3′ |
| E-ACT | 5′-GACTGCGTACCAATTCACT-3′ |
| E-AT | 5′-GACTGCGTACCAATTCAT-3′ |
| M-GT | 5′-GATGAGTCCTGAGTAAGT-3′ |
| M-CAG | 5′-GATGAGTCCTGAGTAACAG-3′ |
| M-CTG | 5′-GATGAGTCCTGAGTAACTG-3′ |
| M-CG | 5′-GATGAGTCCTGAGTAACG-3′ |
| M-GA | 5′-GATGAGTCCTGAGTAAGA-3′ |
| M-TG | 5′-GATGAGTCCTGAGTAATG-3′ |
| M-CA | 5′-GATGAGTCCTGAGTAACA-3′ |
| M-TC | 5′-GATGAGTCCTGAGTAATC-3′ |
| M-CAC | 5′-GATGAGTCCTGAGTAACAC-3′ |
| M-CAT | 5′-GATGAGTCCTGAGTAACAT-3′ |
| M-CTA | 5′-GATGAGTCCTGAGTAACTA-3′ |

**Supplementary Table S2** Primer combinations that generated distinct polymorphism

| Primer combinations generating Polymorphism | Total number of bands | Total number of differential bands | Total number of differential bands due to infection | Up-regulated[1] | Down-regulated[2] |
|---|---|---|---|---|---|
| E-AT/M-GA | 58 | 56 | 22 | 14 | 8 |
| E-AT/M-GT | 32 | 29 | 9 | 0 | 9 |
| E-AT/M-TG | 45 | 42 | 8 | 4 | 4 |
| E-AT/M-TC | 41 | 41 | 14 | 8 | 6 |
| E-AT/M-CA | 37 | 36 | 10 | 7 | 3 |
| E-AT/M-CG | 32 | 29 | 9 | 0 | 9 |
| E-AT/M-CAT | 33 | 32 | 9 | 5 | 4 |
| E-AT/M-CTA | 41 | 40 | 3 | 1 | 2 |
| E-AG/M-GA | 46 | 46 | 19 | 11 | 8 |
| E-AG/M-GT | 31 | 30 | 5 | 1 | 4 |
| E-AG/M-TG | 34 | 32 | 4 | 2 | 2 |
| E-AG/M-TC | 33 | 33 | 6 | 2 | 4 |
| E-AG/M-CA | 49 | 49 | 8 | 4 | 4 |
| E-AG/M-CG | 23 | 21 | 4 | 0 | 4 |
| E-AG/M-CAT | 28 | 28 | 4 | 2 | 2 |
| E-AG/M-CTA | 17 | 17 | 2 | 2 | 0 |
| E-ACT/M-GA | 22 | 21 | 6 | 4 | 2 |
| E-ACT/M-GT | 25 | 23 | 7 | 3 | 4 |
| E-ACT/M-TG | 21 | 21 | 7 | 4 | 3 |
| E-ACT/M-TC | 21 | 21 | 2 | 1 | 1 |
| E-ACT/M-CA | 17 | 16 | 3 | 3 | 0 |
| E-ACT/M-CG | 26 | 26 | 7 | 2 | 5 |
| E-ACT/M-CAT | 14 | 13 | 4 | 4 | 0 |
| E-ACT/M-CTA | 23 | 22 | 6 | 4 | 2 |
| E-AA/M-GA | 22 | 22 | 4 | 2 | 2 |
| E-AA/M-GT | 22 | 20 | 8 | 2 | 6 |
| E-AA/M-TG | 31 | 31 | 7 | 1 | 6 |
| E-AA/M-TC | 11 | 10 | 1 | 1 | 0 |
| E-AA/M-CA | 28 | 28 | 3 | 1 | 2 |
| E-AA/M-CG | 11 | 11 | 5 | 0 | 5 |
| E-AA/M-CAT | 16 | 16 | 6 | 5 | 1 |
| E-AA/M-CTA | 11 | 11 | 2 | 0 | 2 |
| E-AAG/M-GA | 27 | 27 | 15 | 8 | 7 |
| E-AAG/M-TG | 28 | 26 | 10 | 6 | 4 |
| E-AAG/M-CG | 22 | 22 | 5 | 2 | 3 |
| E-AAG/M-CAC | 21 | 20 | 6 | 6 | 0 |
| E-AAG/M-CAT | 24 | 24 | 9 | 8 | 1 |
| E-AAG/M-CAG | 16 | 16 | 6 | 4 | 2 |
| E-AAG/M-CTA | 25 | 25 | 1 | 0 | 1 |
| E-AAG/M-CTG | 37 | 37 | 6 | 1 | 5 |
| E-ACA/M-GA | 7 | 7 | 4 | 3 | 1 |
| E-ACA/M-TG | 14 | 14 | 4 | 2 | 2 |
| E-ACA/M-CG | 10 | 10 | 5 | 1 | 4 |
| E-ACA/M-CAC | 7 | 7 | 3 | 1 | 2 |
| E-ACA/M-CAT | 6 | 6 | 2 | 0 | 2 |
| E-ACA/M-CAG | 4 | 4 | 1 | 1 | 0 |
| E-ACA/M-CTA | 7 | 7 | 1 | 0 | 1 |
| E-ACA/M-CTG | 8 | 8 | 2 | 0 | 2 |
| Total | 1164 | 1133 | 294 | 143 | 151 |
| % of differential TDFs | | | | | 97.33 |
| % of pathogen induced TDFs among total TDFs | | | | | 25.25 |
| % of up-regulated TDFs among pathogen induced TDFs | | | | | 48.63 |
| % of down-regulated TDFs among pathogen induced TDFs | | | | | 51.37 |

[1] Increased expression in infected ICP 8863 compared with infected ICP 2376; [2] less expression or suppression in infected ICP

**Supplementary Table S3** Summary of cDNA-AFLP analysis

| Sl. No. | Condition | No. of TDFs | Remarks | |
|---|---|---|---|---|
| 1. | Total number of TDFs | 1164 | 48 primer combinations generated polymorphism | |
| 2. | Total number of differential TDFs | 1133 | 97.33% of total no of bands | |
| 3. | Total number of differential pathogen-induced TDFs | 294 | 25.25% of total no of bands | |
| 4. | Up-regulated[1] | 143 | 48.63% of pathogen induced TDFs | |
| 5. | Down-regulated[2] | 151 | 51.37% of pathogen induced TDFs | |
| 6. | TDFs excised from gel | 109 | | |
| 7. | TDFs amplified by PCR | 107 | | |
| 8. | TDFs cloned in pGEM-T Easy Vector | 73 | | |
| 9. | Bionformatic analysis of sequences | 58 | | |
| | | | Functions | No. of TDFs |
| 10. | Total TDFs with known function | 42 | Stress/defense | 9 |
| | | | Metabolism | 9 |
| | | | Vascular development | 6 |
| | | | Transcription factors and transcriptional regulation | 5 |
| | | | Transport | 5 |
| | | | DNA repair | 2 |
| | | | Circadian rhythm | 2 |
| | | | Cell component | 1 |
| | | | Signal Transduction | 1 |
| | | | Wound response | 1 |
| | | | Cell wall organiztion | 1 |

[1] Increased expression in infected ICP 8863 compared with infected ICP 2376; [2] less expression or suppression in infected ICP 8863 compared with infected ICP 2376

**Supplementary Table S4** Differential expression of pathogen induced 58 pigeonpea TDFs in infected plants compared to control plants

| Resistant plant | Susceptible plant | No. of TDFs |
|---|---|---|
| Up-regulation | No expression | 13 |
| No expression | Up-regulation | 13 |
| Up-regulation | Down-regulation | 11 |
| Down-regulation | Up-regulation | 6 |
| Constant expression | Down-regulation | 11 |
| Down-regulation | Constant expression | 4 |

**Supplementary Table S5** Primer Sequences used for semi-qRT-PCR

| Gene Name | Primer Sequence (5' - 3') |
|---|---|
| GAPDH | F: ATGGCATTCCGTGTTCCTAC<br>R: CCTTCAACTTGCCCTCTGAC |
| AIG1 | F: TCGCTTTTCTGAAGAGGAAC<br>R: GGACACTCGCGACCTAAATA |
| WOX4 | F: GGAAGATAGTCCGTACAAGAAG<br>R: CCAGAGTTCTATGTTCCTCCT |
| MYB46 | F: CTTCTGGTGGTGGATTCTTC<br>R: ATCTGTGTTGCATGTGCTCT |
| WRKY70 | F: GCCATCAAATTCCAAACCAC<br>R: GAAACAGGAGCCTGTGGAAG |
| CLE24 | F: ATGGTGTAATGGGTTTGGAC<br>R: TCCAAGATCAACAAGAAGCA |
| SUA | F: TACAACTCTTGCCCAGGCTG<br>R: CCTCTTCCTGAGCCCCTTAT |
| IMPORTIN | F: TTCCAATAATGCATGACCAG<br>R: TAAAGGGAGGCAACTGATTC |
| ABCG11 | F: CGTGCATACCACAAAGGTAA<br>R: GGTTCGCTGCTCATATTTTT |

(F: Forward primer, R: Reverse primer)
